# Cortisol affects macrophage polarization by inducing miR-143/145 cluster to reprogram glucose metabolism and by promoting TCA cycle anaplerosis

**DOI:** 10.1016/j.jbc.2024.107753

**Published:** 2024-09-10

**Authors:** Amod Sharma, Kunwar Somesh Vikramdeo, Sarabjeet Kour Sudan, Shashi Anand, Sachin Kumar Deshmukh, Ajay Pratap Singh, Seema Singh

**Affiliations:** 1 Cancer Center and Research Institute, University of Mississippi Medical Center, Jackson, Mississippi, USA; 2 Department of Cell and Molecular Biology, University of Mississippi Medical Center, Jackson, Mississippi, USA; 3 Mitchell Cancer Institute, University of South Alabama, Mobile, Alabama, USA; 4 Department of Pathology, University of South Alabama, Mobile, Alabama, USA

**Keywords:** macrophage, polarization, cortisol, miRNA-143-3p, miR-145-5p

## Abstract

Chronic stress can have adverse consequences on human health by disrupting the hormonal balance in our body. Earlier, we observed elevated levels of cortisol, a primary stress hormone, and some exosomal microRNAs in the serum of patients with breast cancer. Here, we investigated the role of cortisol in microRNA induction and its functional consequences. We found that cortisol induced the expression of miR-143/145 cluster in human monocyte (THP1 and U937)-derived macrophages but not in breast cancer cells. *In silico* analysis identified glucocorticoid-response element in the upstream *CARMN* promoter utilized by the miR-143/145 cluster. Enhanced binding of glucocorticoid-receptor (GR) upon cortisol exposure and its regulatory significance was confirmed by chromatin-immunoprecipitation and promoter-reporter assays. Further, cortisol inhibited IFNγ-induced M1 polarization and promoted M2 polarization, and these effects were suppressed by miR-143-3p and miR-145-5p inhibitors pretreatment. Cortisol-treated macrophages exhibited increased oxygen-consumption rate (OCR) to extracellular-acidification rate (ECAR) ratio, and this change was neutralized by functional inhibition of miR-143-3p and miR-145-5p. *HK2* and *ADPGK* were confirmed as the direct targets of miR-143-3p and miR-145-5p, respectively. Interestingly, silencing of *HK2* and *ADPGK* inhibited IFNγ-induced M1 polarization but failed to induce M2 polarization, since it suppressed both ECAR and OCR, while OCR was largely sustained in cortisol-treated M2-polarized macrophages. We found that cortisol treatment sustained OCR by enhancing fatty acid and glutamine metabolism through upregulation of *CPT2* and *GLS*, respectively, to support M2 polarization. Thus, our findings unfold a novel mechanism of immune suppression by cortisol and open avenues for preventive and therapeutic interventions.

Macrophages are an indispensable component of innate immunity. They acquire different forms in different locations, such as microglia in the central nervous system, Kupfer cells in the liver, histiocytes in connective tissue, alveolar macrophages in lungs, and osteoclasts in bone tissues (1, 2). Macrophages show high plasticity and gain distinct properties according to their polarization state. The classically activated or M1 polarized macrophages are phagocytic, produce pro-inflammatory cytokines, and thus initiate an immune response. In contrast, alternatively activated anti-inflammatory macrophages (M2 polarized) resolve the inflammation, help in tissue repair, and are considered immunosuppressive (3, 4, 5, 6). The polarization state of the macrophage is governed by external stimuli and plays an essential role in maintaining the homeostasis and pathobiology of several benign and malignant diseases (7).

Chronic stress is associated with various health problems, including cardiovascular diseases, obesity, diabetes, and cancer (8). Stress activates the hypothalamic-pituitary-adrenal axis and the sympathetic nervous system leading to the production of stress hormones (9, 10). Cortisol is the primary stress hormone released as a fight-or-flight response. It increases blood glucose levels and enhances glucose usage by the brain while cutting down unnecessary functions (11, 12). Under normal circumstances, blood cortisol levels follow a circadian rhythm with higher levels during the daytime and very low or undetectable at midnight (13). Chronic stress breaks this cycle affecting our sleep and other physiological functions (14, 15). Chronic stress has also been associated with poor immune function and is a risk factor for cancer development and aggressiveness (16, 17). High plasma cortisol levels are reported in patients with metastatic breast cancer (BC) than those with early-stage disease, promote glucocorticoid receptor (GR) activity at distant metastatic sites and support cancer cell colonization (18, 19). In a recent population-based study, we have also observed elevated levels of cortisol in the serum of patients with BC than non-cancer participants along with the increased presence of certain micro-RNAs in the serum exosomes (20). Interestingly, cortisol levels were higher in African American (AA) women than in Caucasian American (CA) women regardless of cancer diagnosis.

In the present study, we examined the effect of cortisol on the expression of miRNAs that were differentially present in the serum exosomes of BC patients and delineated underlying regulatory mechanisms and functional consequences. Cortisol induced the expression of miR-143-3p/miR-145 cluster in macrophages but not in BC cells. Further, cortisol transcriptionally upregulated the expression of the miR-143-3p/miR-145 cluster by promoting the GR binding to the *CARMN* promoter. In addition, it also suppressed the IFNγ-mediated induction of M1 polarization and promoted their M2 polarization by altering the glucose metabolism. Hexokinase 2 (*HK2*) and ADP-dependent glucokinase (*ADPGK*) were characterized as direct targets of miR-143-3p and miR-145-5p, respectively, which mediated their effects on macrophage polarization. Interestingly, cortisol also induced the expression of carnitine palmitoyltransferase 2 (*CPT2*) and glutaminase (*GLS*), key enzymes in fatty acid oxidation and glutaminolysis to maintain oxidative phosphorylation (OXPHOS) in M2 macrophages. These data reveal a novel mechanism of immunosuppressive action of cortisol that could potentially explain race-associated poor clinical outcomes in BC patients.

## Results

### Cortisol induces miR-143-3p expression in macrophages

**Figure 1:**
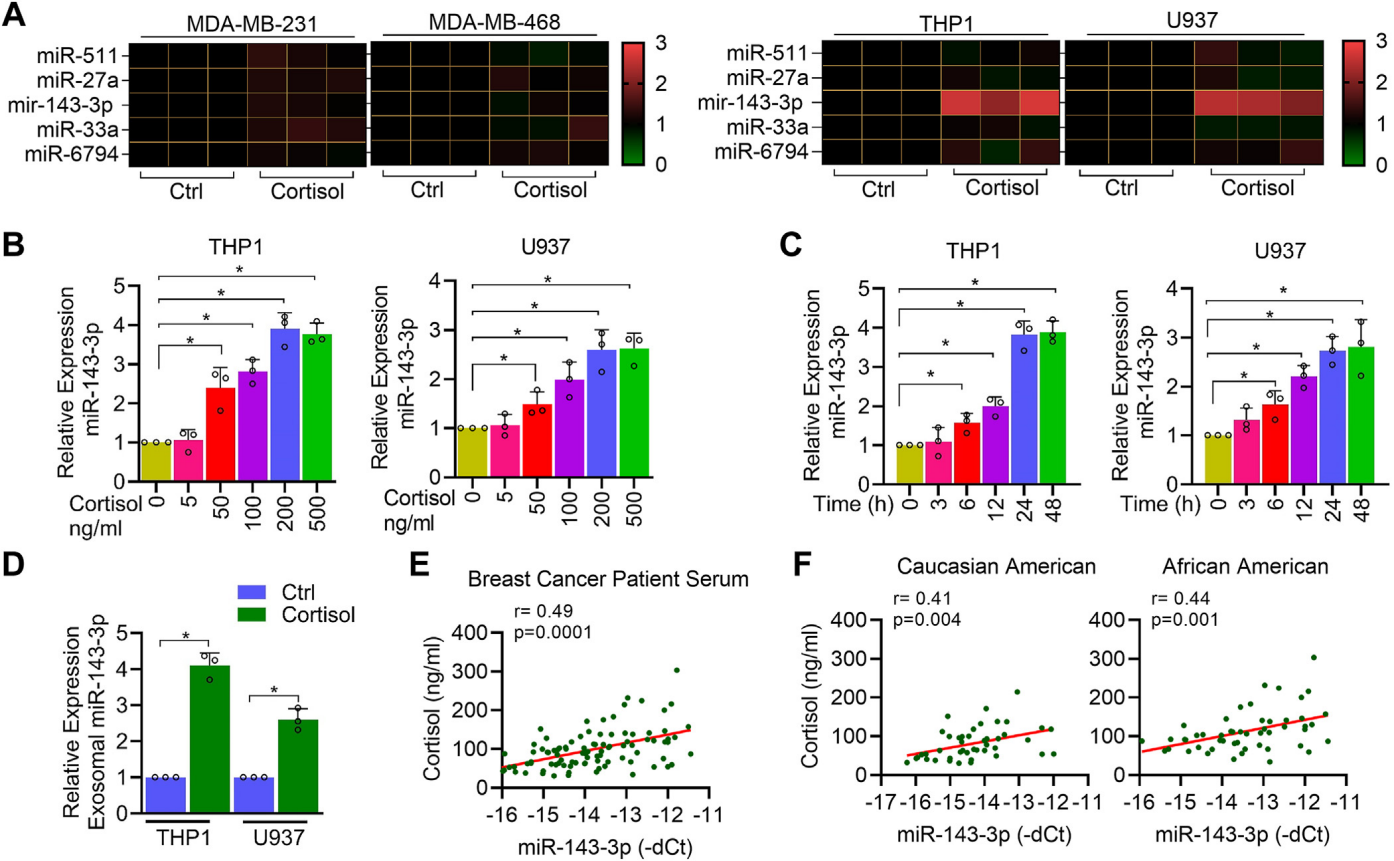
**Cortisol treatment in macrophages induces miR-143-3p expression.***A*, Heat map showing expression profiling of miR-511, miR-27a, miR-143-3p, miR-33a and miR-6794 in breast cancer cell lines (MDA-MB-231 and MDA-MB-468) and monocyte cell lines (THP1 and U937) -derived macrophages treated with 200 ng/ml cortisol for 24 h. *B*, qRT-PCR data showing dose-dependent induction of miR-143-3p by cortisol in THP1- and U937-derived macrophages treated for 24 h. *C*, qRT-PCR data showing time-dependent induction of miR-143-3p upon treatment with 200 ng/ml cortisol for varying time intervals in THP1- and U937-derived macrophages. *D*, the relative fold difference of miR-143-3p levels in exosomes secreted from THP1- and U937-derived macrophages following treatment with cortisol (200 ng/ml) for 48 h cortisol, as determined by qRT-PCR. *E*, correlation of serum cortisol levels estimated by ELISA with serum exosomal miR-143-3p in breast cancer patients (each dot represents a patient; n = 97). Pearson correlation analysis was performed to derive Pearson correlation coefficient (r). *F*, correlation of serum cortisol and exosomal miR-143-3p in Caucasian American (CA) and African American (AA) patients (each dot represents a patient; CA n = 46, AA n = 51). Data were analyzed for statistical significance by One-Way ANOVA with Tukey’s multiple comparisons test (*B* and *C*) or unpaired Student’s *t* test (*D*). ∗*p* < 0.05.

As in a parallel study, we identified a higher abundance of cortisol and microRNAs (miR-143-3p, miR-511, miR-27a, miR-33a, and miR-6794) in exosomes derived from BC patient serum than that from non-cancer subjects (20), we interrogated if cortisol played a role in regulating the expression of these microRNAs. For this, we treated BC cells (MDA-MB-231 and MDA-MB-468) and monocyte cell lines (THP1 and U937) -derived macrophages with two physiologically relevant doses (100 and 200 ng/ml) of cortisol. We observed that miR-143-3p was upregulated in macrophages at both doses but not in the BC cells (Figs. 1*A* and S1*A*). We next treated THP1 and U937 macrophages with different doses (0–500 ng/ml) of cortisol and observed a dose-dependent upregulation of miR-143-3p with a maximum increase (THP1, 3.8-fold; U937, 2.6-fold) at 200 ng/ml of cortisol (Fig 1*B*). Similarly, a significant time-dependent (0–48 h) increase in expression of miR-143-3p was also detected upon cortisol treatment (200 ng/ml) with a maximum increase of 3.7- and 2.8-fold in THP1 and U937 macrophages, respectively (Fig. 1*C*). Since we found miR-143-3p in the serum exosomes of BC patients, we examined its abundance in macrophage-derived exosomes after cortisol treatment. Exosomes were isolated from the treated cells and their authenticity was confirmed by western blotting for exosomal markers, CD9 and CD63 (Fig. S1*B*). We observed a significantly higher presence of miR-143-3p in the macrophage-derived exosomes (THP1, 4.0-fold; U937, 2.6-fold) after cortisol treatment (Fig. 1*D*). Using the dataset from our parallel study (20), we also analyzed the correlation between cortisol and exosomal miR-143-3p from BC patients and found a significant positive association (r = 0.49; *p* = 0.0001) (Fig. 1*E*). Interestingly, in a race-specific comparison, a slightly better correlation was observed in AA in patients with BC (r = 0.44; *p* = 0.001) than in CA in patients with BC (r = 0.41; *p* = 0.004) (Fig. 1*F*).

### Cortisol regulates transcription of CARMN/miR-143/miR-145 cluster through direct binding of GRα to *CARMN* promoter

**Figure 2:**
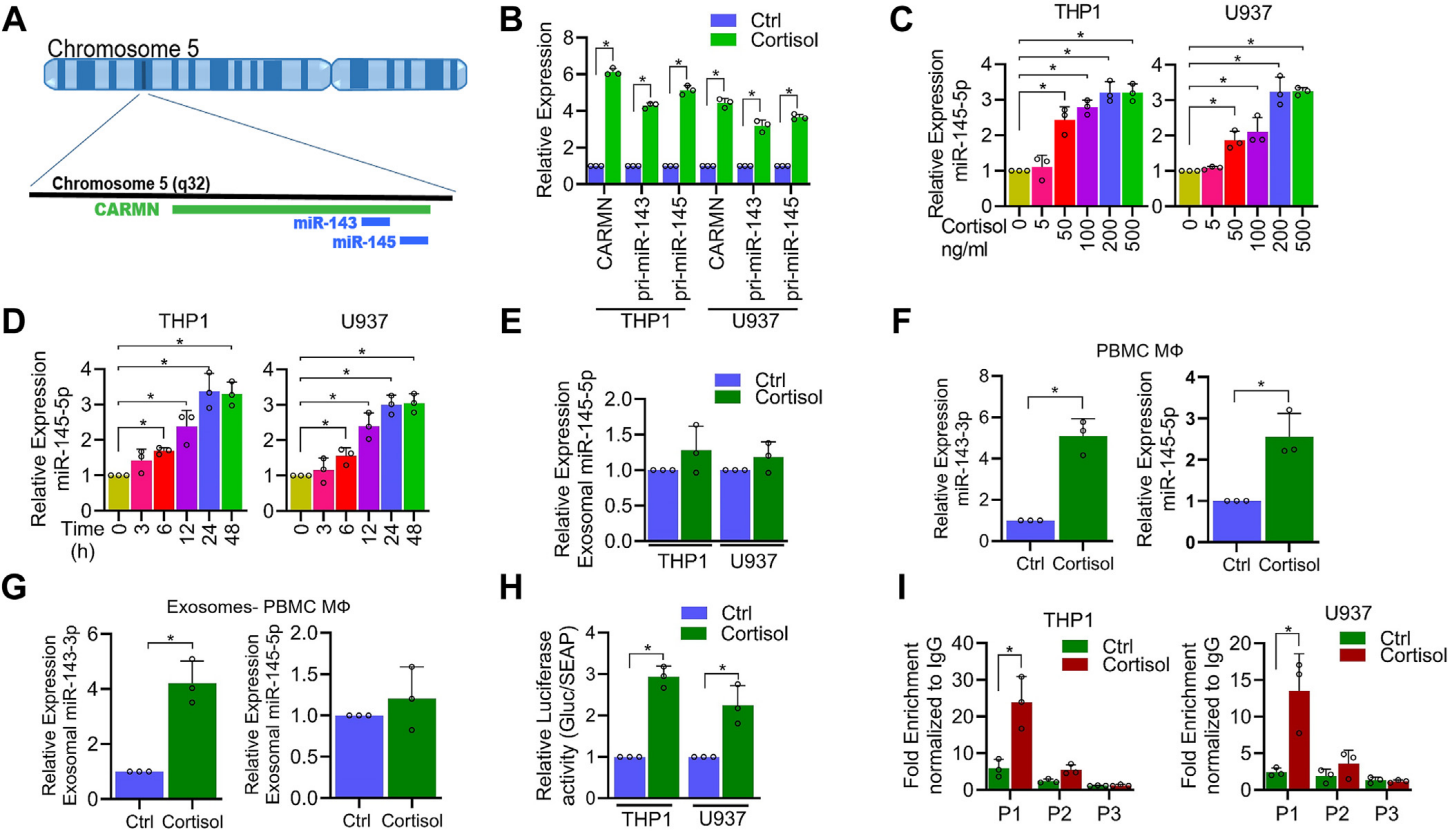
**Direct binding of cortisol receptor GRα regulates expression of CARMN/miR-143/miR-145 cluster.***A*, Graphical illustration showing the position of long non-coding RNA CARMN, miR-143 and miR-145 on chromosome 5. *B*, relative RNA expression of CARMN, pri-miR-143, and pri-miR-145 in cortisol (200 ng/ml) -treated THP1- and U937-derived macrophages as analyzed by qRT-PCR. *C*, Dose-dependent relative induction of miR-145-5p in THP1- and U937-derived macrophages treated with increasing concentration of cortisol for 24 h as analyzed by qRT-PCR. *D*, Time-dependent induction of miR-145-5p in THP1- and U937-derived macrophages treated with 200 ng/ml cortisol as analyzed by qRT-PCR. *E*, relative fold change in exosomal miR-145-5p levels isolated from THP1- and U937-derived macrophages treated with cortisol (200 ng/ml) for 48 h. *F*, qRT-PCR analysis showing induction of miR-143-3p and miR-145-5p in PBMC-derived macrophages treated with cortisol (200 ng/ml) for 24 h. *G*, relative fold difference in exosomal miR-143-3p and miR-145-5p isolated from PBMC-derived macrophages following treatment with cortisol (200 ng/ml) for 48 h. *H*, luciferase promoter reporter assay exhibiting relative *CARMN* promoter activity in THP1- and U937-derived macrophages following treatment with cortisol (200 ng/ml). *I*, ChIP assay showing fold enrichment of GRα binding at putative binding regions (P1, P2, and P3) in *CARMN* promoter following treatment with cortisol (200 ng/ml). Data were analyzed for statistical significance by unpaired Student’s *t* test (*B*, *E*–*I*) or One-Way ANOVA with Tukey’s multiple comparisons test (*C* and *D*). ∗*p* < 0.05.

To examine the mechanistic basis of cortisol-induced miR-143-3p upregulation, we first searched the genomic location of miR-143-3p using the UCSC genome browser. We located it on chromosome 5q32 within a host long non-coding (lnc) RNA *CARMN* (cardiac mesoderm enhancer-associated noncoding RNA), along with another miRNA, miR-145 (Fig. 2*A*). To confirm the regulation of the CARMN/miR-143/miR-145 cluster by cortisol, we treated macrophages and found that cortisol significantly induced the expression of *CARMN*, pri-miR-143, and pri-miR-145 in both THP1and U937 macrophages (Fig. 2*B*). Likewise, we also observed an upregulation of miR-145-5p upon cortisol treatment in a dose (0–500 ng/ml) and time (0–48 h) dependent manner (Fig. 2, *C* and *D*). The expression of miR-143-5p and miR-145-3p was negligible in THP1 and U937 macrophages and did not significantly change even after cortisol treatment (Table S1*A*). Since miR-143-3p was present at higher levels in the exosomes secreted from cortisol-treated macrophages, we also examined the levels of exosomal miR-145-5p. No significant increase in exosomal miR-145-5p levels was; however, observed after cortisol treatment in both macrophage cell lines (Fig. 2*E*). These findings were validated in human PBMC-derived macrophages and their exosomal fractions after confirming the authenticity based on markers (CD9 and CD63) expression (Fig. S2*A*). Following cortisol treatment, a significant increase in the levels of both miR-143-3p and miR145-5p was observed in human PBMC-derived macrophages (Fig. 2*F*) but only miR-143-3p levels increased in the exosomal fraction (Fig. 2*G*). To confirm the direct transcription of the CARMN/miR-143-3p/miR-145-5p cluster by cortisol, we performed the promoter-reporter assay by transfecting THP1- and U937-derived macrophages with a luciferase vector containing *CARMN* promoter region. We found 1.5 to 3.0-fold induction in luciferase activity upon treatment with cortisol compared to control cells (Fig. 2*H*). To examine the possibility of direct regulation through GRα (cortisol receptor), we performed *in silico* analysis to predict its binding site(s) in the promoter region using the JASPAR database (Fig. S2*B*). Three binding sites of GRα were predicted of which one was present in a far distant region (P1: -562 to -727; P2: -1282 to -1441; and P3: -4797 to -4981) (Fig. S2*C*). ChIP assay was performed using specific primer sets to confirm GRα binding. The highest binding of GRα was observed in the P1 region (˃15 folds) followed by the P2 region (∼5 folds), while a negligible enrichment was reported at the distal P3 site (Fig. 2*I*). To further confirm that the binding of GRα at the P1 site was essential for the transcriptional activity, we mutated this region in the *CARMN* promoter at four base pairs (Fig. S3*A*) and performed the promoter-reporter assay. We observed that the induction of luciferase activity by cortisol was abrogated in cells transfected with the P1 mutant luciferase promoter-reporter (Fig. S3*B*).

### Inhibition of IFNγ-induced M1 polarization and induction of M2 polarization of macrophages by cortisol is mediated through miR-143-3p and miR-145-5p

**Figure 3:**
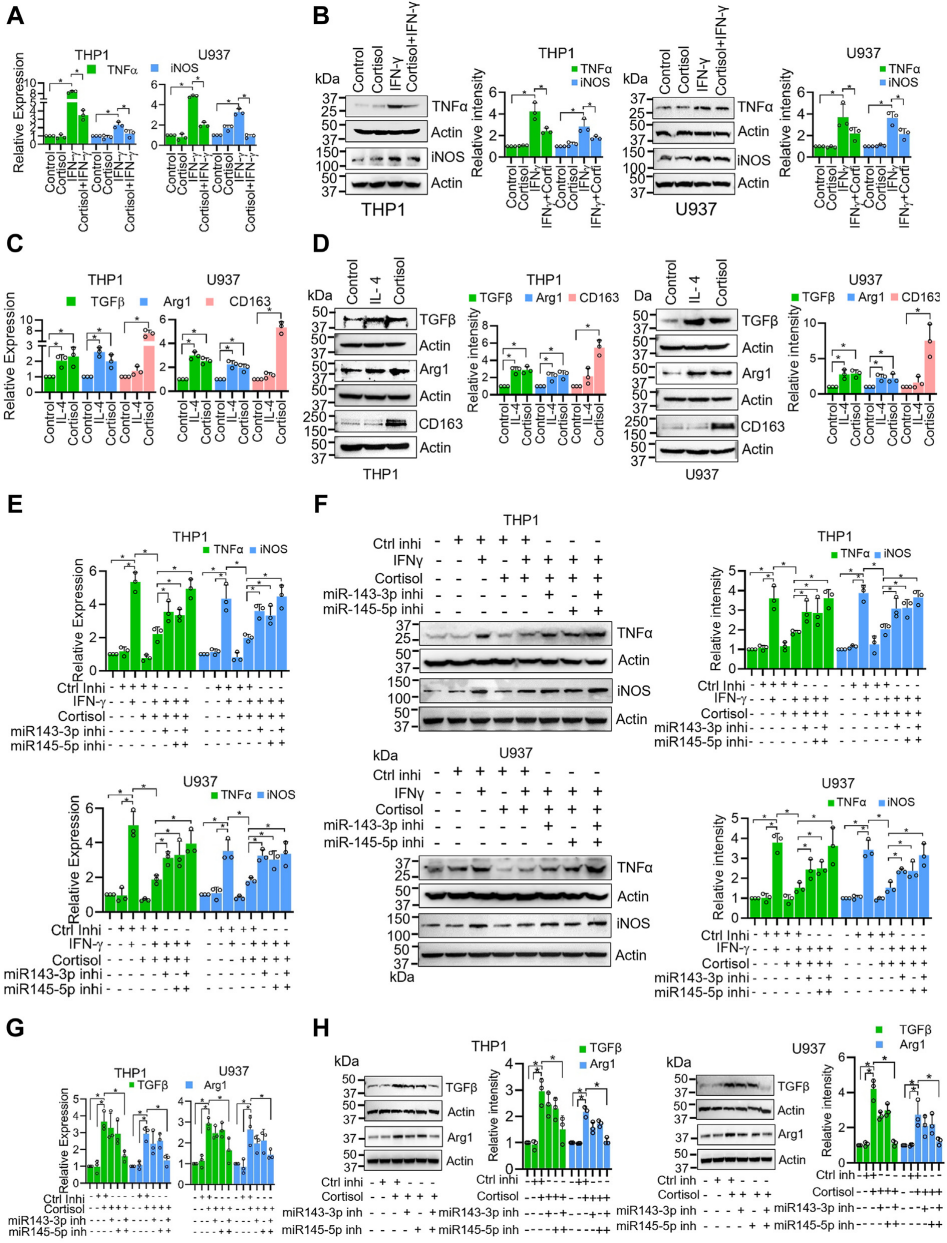
**Cortisol-mediated M1 inhibition and M2 induction is facilitated through miR-143-3p and miR-145-5p.***A*–*B*, Inhibition of IFNγ (50 ng/ml)-induced M1 polarization by cortisol (200 ng/ml) pretreatment in THP1- and U937-derived macrophages. Expression of M1 markers (TNFα and iNOS) was analyzed by qRT-PCR (*A*) or western blotting (*B*) following 48 h of IFNγ treatment. *C*–*D*, Induction of M2 polarization markers (TGFβ, Arg1 and CD163) in THP1- and U937-derived macrophages following cortisol (200 ng/ml) or IL-4 (50 ng/ml) treatment for 48 h as analyzed by qRT-PCR (*C*) and western blotting (*D*). *E*–*F*, rescue of cortisol-mediated inhibition of IFNγ-induced M1 polarization by inhibitors of miR-143-3p and miR-145-5p as analyzed by qRT-PCR (*E*) and western blotting (*F*). *G*–*H*, inhibition of cortisol-induced M2 polarization by inhibitors of miR-143-3p and miR-145-5p as analyzed by qRT-PCR (*G*) and western blotting (*H*). Bars in panels *B*, *D*, *F*, and *H* represent quantitative data obtained by densitometry of band intensity in three biological replicates. Data were analyzed for statistical significance by One-Way ANOVA with Tukey’s multiple comparisons test. ∗*p* < 0.05.

Macrophages are broadly subdivided into two categories *viz*. M1 (immunostimulatory) and M2 (immunosuppressive) types and their differential polarization depend on their stimuli. The M1 and M2 macrophage polarization markers were used in line with the established literature (7, 21, 22, 23, 24, 25). To study the immunosuppressive effect of cortisol, we treated THP1-, U937- and PBMC-derived macrophages with cortisol and examined the expression of M2 markers. In addition, we also pre-treated these macrophages with cortisol prior to stimulation with interferon-gamma (IFNγ), a known inducer of M1 polarization. We observed that IFNγ treatment induced the expression of M1 markers, TNFα and iNOS, at both RNA and protein levels, which was abrogated in cells pretreated with cortisol (Figs. 3, *A*–*B* and S4*A*). Cortisol treatment also led to an induced expression of M2 markers (TGFβ, Arg1, and CD163). Interestingly, although the induction of transforming growth factor beta (TGFβ) and Arginase 1 (Arg1) by cortisol was comparable to that induced by Interleukin 4 (IL-4), a known inducer of M2 polarization; induction of CD163 was far greater in cortisol-treated macrophages (Figs. 3, *C*–*D*; S4*B*). These observations were further confirmed by flow cytometry analysis using CD86 (M1 surface marker) and CD163 (M2 surface marker) (Fig. S5, *A*–*B*).

To examine the role of miR-143-3p and miR-145-5p in cortisol-induced macrophage polarization, we pretreated them with miRNA inhibitors prior to cortisol treatment. We found that macrophage pretreatment with miR-143-3p or miR-145-5p inhibitors abrogated cortisol-mediated inhibition of M1 polarization markers both at RNA and protein levels (Figs. 3, *E*–*F*; S6*A*). Further, we observed inhibition of cortisol-induced M2 polarization in macrophages pre-treated with miR-143-3p and miR-145-5p inhibitors (Figs. 3, *G*–*H*; S6*B*). A more potent inhibition was observed when miR-143-3p and miR-145-5p inhibitors were used in combination. These findings were also validated by flow cytometry for M1 and M2 surface markers (CD86 and CD163, respectively) (Fig. S7, *A*–*D*). These data show that miR-143-3p and miR-145-5p mediate the effect of cortisol on macrophage polarization.

### miR-143-3p and miR-145-5p mediate cortisol-induced increase in OCR to ECAR ratio

**Figure 4:**
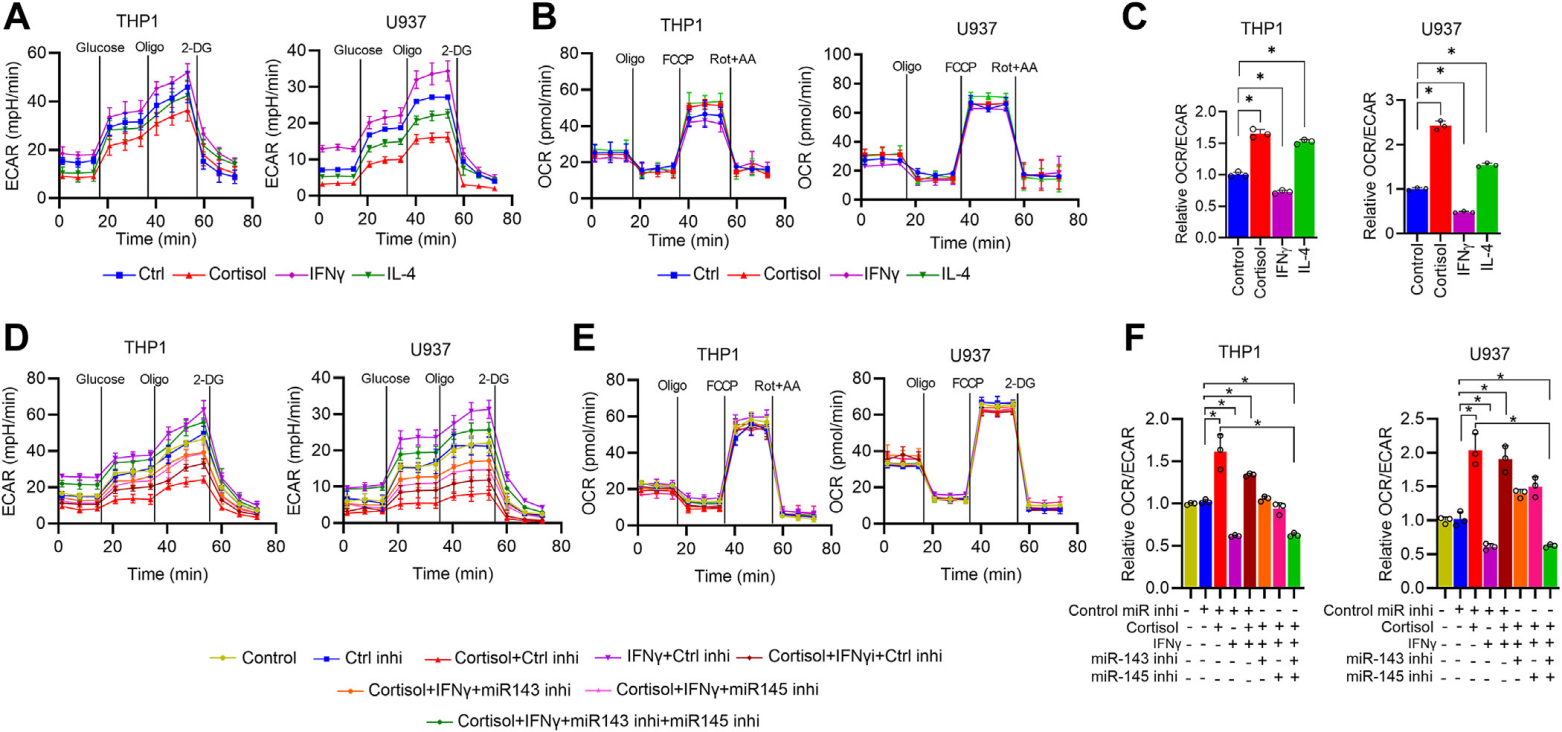
**Cortisol increases OCR to ECAR ratio in macrophages through miR-143-3p and miR-145-5p.***A*–*C*, THP1- and U937-derived macrophages were treated with cortisol (200 ng/ml) or IFNγ (50 ng/ml) or IL-4 (50 ng/ml) for 48 h and analyzed for ECAR (*A*), OCR (*B*) and OCR to ECAR ratio (*C*). *D*–*F*, THP1- and U937-derived macrophages were treated with control or miR-143-3p and miR-145-5p inhibitors along with cortisol prior to 48 h treatment of IFNγ and analyzed for ECAR (*D*), OCR (*E*), and OCR to ECAR ratio (*F*). Data were analyzed for statistical significance by One-Way ANOVA with Tukey’s multiple comparisons test. ∗*p* < 0.05.

It has been shown that M1 macrophages have a higher reliance on glycolysis, while M2 macrophages are more inclined toward OXPHOS for their energy requirements (26, 27). Therefore, to examine the impact of cortisol treatment on macrophage metabolism, we performed extracellular acidification rate (ECAR) and oxygen consumption rate (OCR) measurements. We found that IFNγ treatment increased the ECAR, which was suppressed upon cortisol treatment in THP1- and U937-derived macrophages (Fig. 4*A*). We also performed L-lactate assay, which is another method to measure glycolysis, and observed similar results (Fig. S8*A*). We then analyzed the OXPHOS rate in the macrophages by performing an OCR assay. We observed that OXPHOS remained largely unchanged in IFNγ, IL-4, or cortisol-treated cells compared to control (Fig. 4*B*). Further, by analyzing the ratio for the OCR/ECAR, we found that the ratio was reduced in IFNγ-treated macrophages showing M1 type phenotype, while significantly increased in IL-4 and cortisol-treated macrophages showing M2 type phenotype (Fig. 4*C*). To check if these changes in metabolic profile by cortisol treatment were regulated by miR-143-3p and miR-145-5p, we transfected the cells with miRNA inhibitors prior to cortisol treatment. We observed that the suppression of glycolysis by cortisol was rescued when the macrophages were pretreated with a combination of miR-143-3p and miR-145-5p inhibitors (Fig. 4, *D* and *E*). Further, the OCR/ECAR ratio in the cells treated with combination of both miR-inhibitors was comparable to that of IFNγ only treated cells. (Fig. 4*F*). These data show that metabolic changes caused by cortisol were mediated through the upregulation of miR-143-3p and miR-145-5p. Since the ECAR was decreased in cortisol-treated macrophages but the OCR was sustained, we investigated if they shifted their dependency on alternative metabolite sources to feed the tricarboxylic acid cycle (TCA) cycle. A fuel flexibility assay was performed to examine the OCR rate using glucose, fatty acid, and glutamine as fuel sources. The data show that IFNγ-treated macrophages were more dependent on glucose. At the same time, cortisol- and IL-4-treated became more dependent on fatty acid and glutamine as fuel sources for the TCA cycle (Fig. S8*B*).

### miR-143-3p and miR-145-5p repress the expression of *HK2* and *ADPGK*, respectively, by directly binding to their 3ˈ UTRs

**Figure 5:**
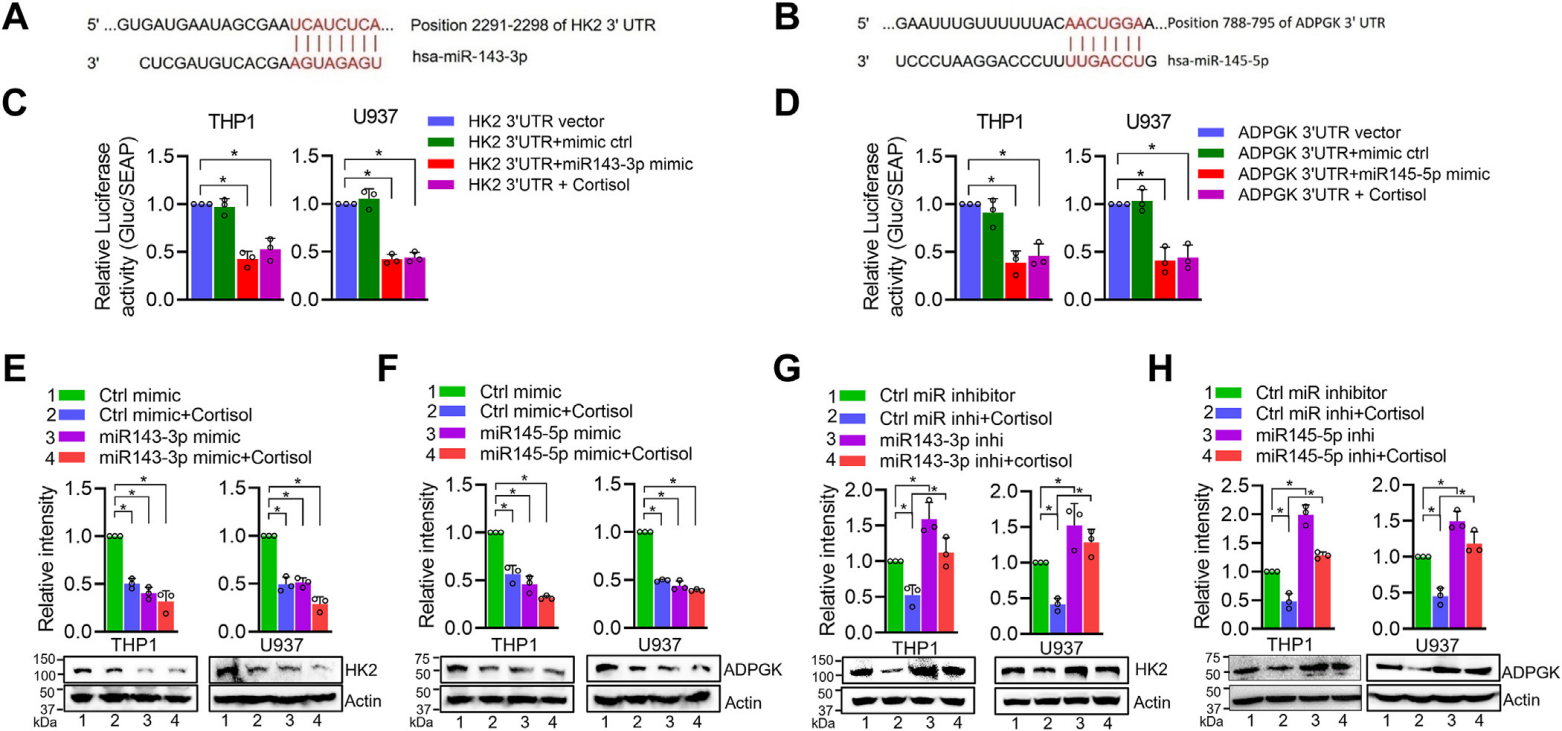
**miR-143-3p and miR-145-5p target *HK2* and *ADPGK* respectively.***A–B*, graphical illustration showing the binding sequence of miR-143-3p and miR-145-5p in 3ʹ UTR regions of HK2 mRNA (*A*) and ADPGK mRNA (*B*), respectively. *C*–*D*, dual luciferase assay showing a reduction in luciferase activity resulting from targeting of *HK2* (*C*) and *ADPGK* (*D*) 3ʹ UTRs upon treatment with miR-143-3p mimic and miR-145-5p respectively or cortisol (200 ng/ml) for 24 h in THP1- and U937-derived macrophages. *E*-*F*, Western blot data showing reduced expression of HK2 (*E*) and ADPGK (*F*) at the protein level in THP1- and U937-derived macrophages treated with cortisol or miR-143-3p or miR-145-5p mimics alone and in combination for 24 h. *G*–*H*, Western blot analysis showing rescued expression of HK2 (*G*) and ADPGK (*H*) at the protein levels in THP1- and U937-derived macrophages pretreated with inhibitors of miR-143-3p or miR-145-5p prior to treatment with cortisol for 24 h. Bars in panels *E*–*H* represent quantitative data obtained by densitometry of band intensity in three biological replicates. Data were analyzed for statistical significance by One-Way ANOVA with Tukey’s multiple comparisons test. ∗*p* < 0.05.

Since we observed the effect of the miR-143-3p and miR-145-5p on the ECAR, we searched their target genes essential for glycolysis. Using TargetScan and miRDB, we found hexokinase-2 (*HK2*) and ADP-dependent glucokinase (*ADPGK*) as potential target genes of miR-143-3p and 145-5p, respectively (Fig. 5, *A* and *B*). It is also interesting to note that the classically-activated macrophages (M1 type) show increased expression of *HK2* and *ADPGK* (27, 28, 29). We cloned the 3′ untranslated region (3′ UTR) of *HK2* and *ADPGK* in a luciferase reporter plasmid and transfected them in macrophages prior to cortisol or miRNA mimics treatment. The data show a significant reduction in luciferase activity in macrophages transfected with miRNA mimics suggesting the functional binding of miR-143-5p and miR-145-3p mimics at *HK2* and *ADPGK* 3ˈ UTR regions, respectively (Fig. 5, *C* and *D*). We also observed that *HK2* and *ADPGK* expression was reduced in macrophages treated with the miR-143-3p and miR-145-5p mimics, respectively or by cortisol (Fig. 5, *E* and *F*). In contrast, we observed an increased expression of *HK2* and *ADPGK* when the cells were treated with the miR-143-3p and miR-145-5p inhibitors (Fig. 5, *G* and *H*). These findings confirm *HK2* and *ADPGK* as the direct targets of miR-143-3p and miR-145-5p, respectively, to regulate glycolysis negatively.

### Knockdown of *HK2* and *ADPGK* inhibits IFNγ induces M1 polarization but does not induce M2 polarization

**Figure 6:**
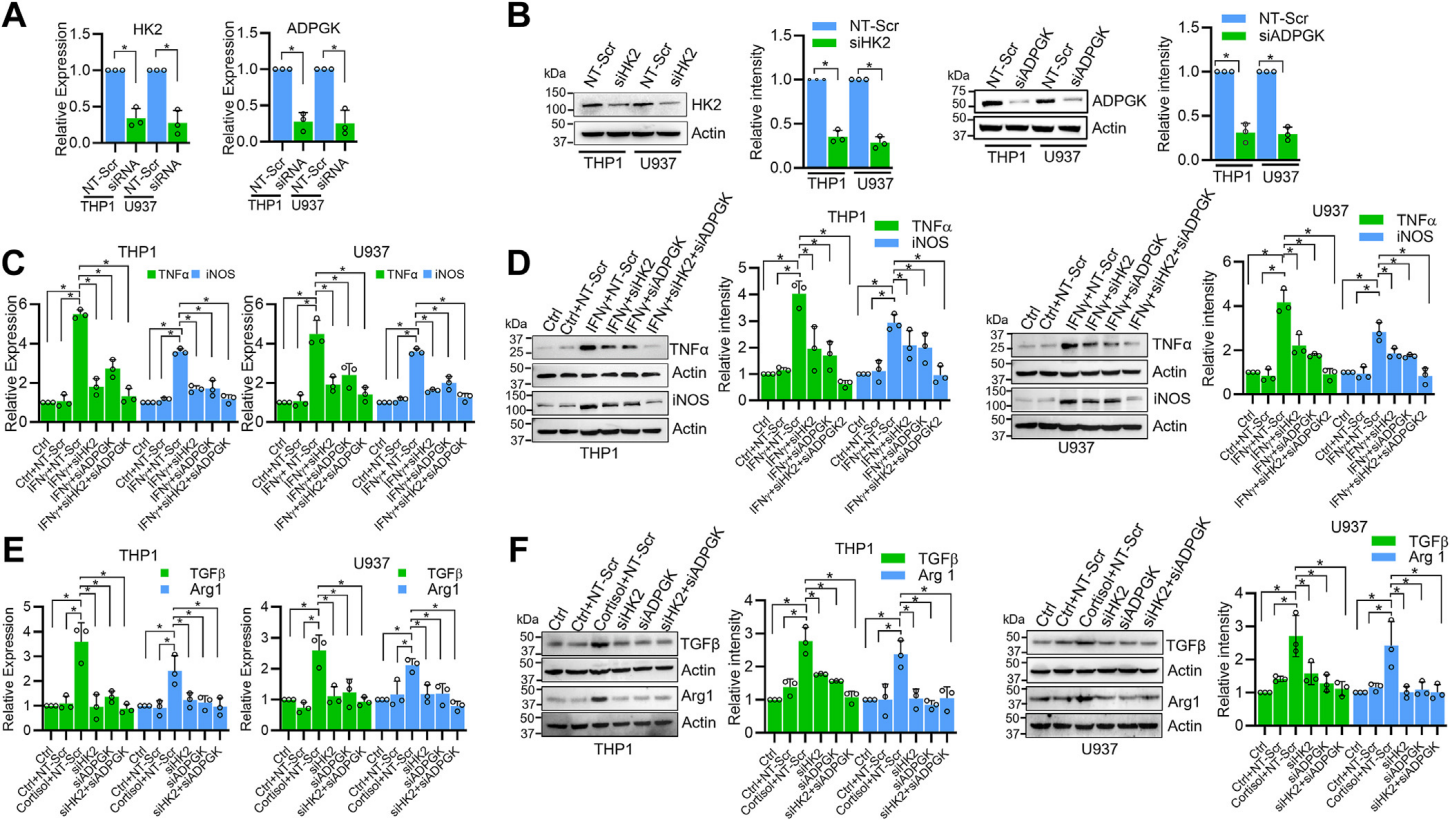
***HK2* and *ADPGK* knockdown inhibits M1 polarization but do not induce M2 polarization.***A*–*B*, siRNA-mediated knockdown of *HK2* and *ADPGK* in THP1- and U937-derived macrophages as analyzed by qRT-PCR (*A*) and western blotting (*B*). *C*–*D*, Inhibition of IFNγ-induced M1 polarization markers (TNFα and iNOS) with siRNA-mediated knockdown of *HK2* and *ADPGK* in THP1- and U937-derived macrophages as analyzed by qRT-PCR (*C*) and Western blotting (*D*). *E*-*F*, relative differences in mRNA (*E*) and protein (*F*) levels of M2 polarization markers (TGFβ and Arg1) in THP1- and U937-derived macrophages upon 48 h treatment with 200 ng/ml cortisol, siHK2 or siADPGK or both siRNAs as analyzed by qRT-PCR and Western blot analyses, respectively. Bars in panels *B*, *D*, and *F* represent quantitative data obtained by densitometry of band intensity in three biological replicates. Quantitative data were analyzed by unpaired Student’s *t* test (*A* and *B*) or one-way ANOVA with Tukey’s multiple comparisons test (*C*–*F*). ∗*p* < 0.05.

To further ensure that *HK2* and *ADPGK* genes are important for the M1 polarization of macrophages, we knockdown their expression by respective siRNAs (Fig. 6, *A* and *B*). We observed that the knockdown of either *HK2* or *ADPGK* or both resulted in significant inhibition of M1 macrophage polarization induced by IFNγ treatment (Fig. 6, *C* andd *D*). We next studied the effect of the knockdown of *HK2* and *ADPGK* on cortisol-induced M2 polarization of macrophages. We found that the treatment of cortisol induced M2 polarization; however, the knockdown of *HK2* or *ADPGK* or both failed to cause M2 polarization of macrophages (Fig. 6, *E* and *F*). When we checked glycolysis rate (ECAR) and OXPHOS rate (OCR) after knockdown of *HK2* and *ADPGK*, we observed that downregulation of *HK2* or *ADPGK* or both resulted in a reduction of glycolysis as well as OXPHOS rate which was expected. In cortisol-treated cells, the reduction in glycolysis did not show a proportionate decrease in the OXPHOS rate (Fig. S9, *A* and *B*). This indicates that the TCA cycle in cortisol-treated macrophages is supported by alternated pathways.

### Cortisol-induced M2 polarization involves sustained oxidative phosphorylation by enhanced fatty acid and glutamine metabolism through upregulation of *CPT2* and *GLS*

**Figure 7:**
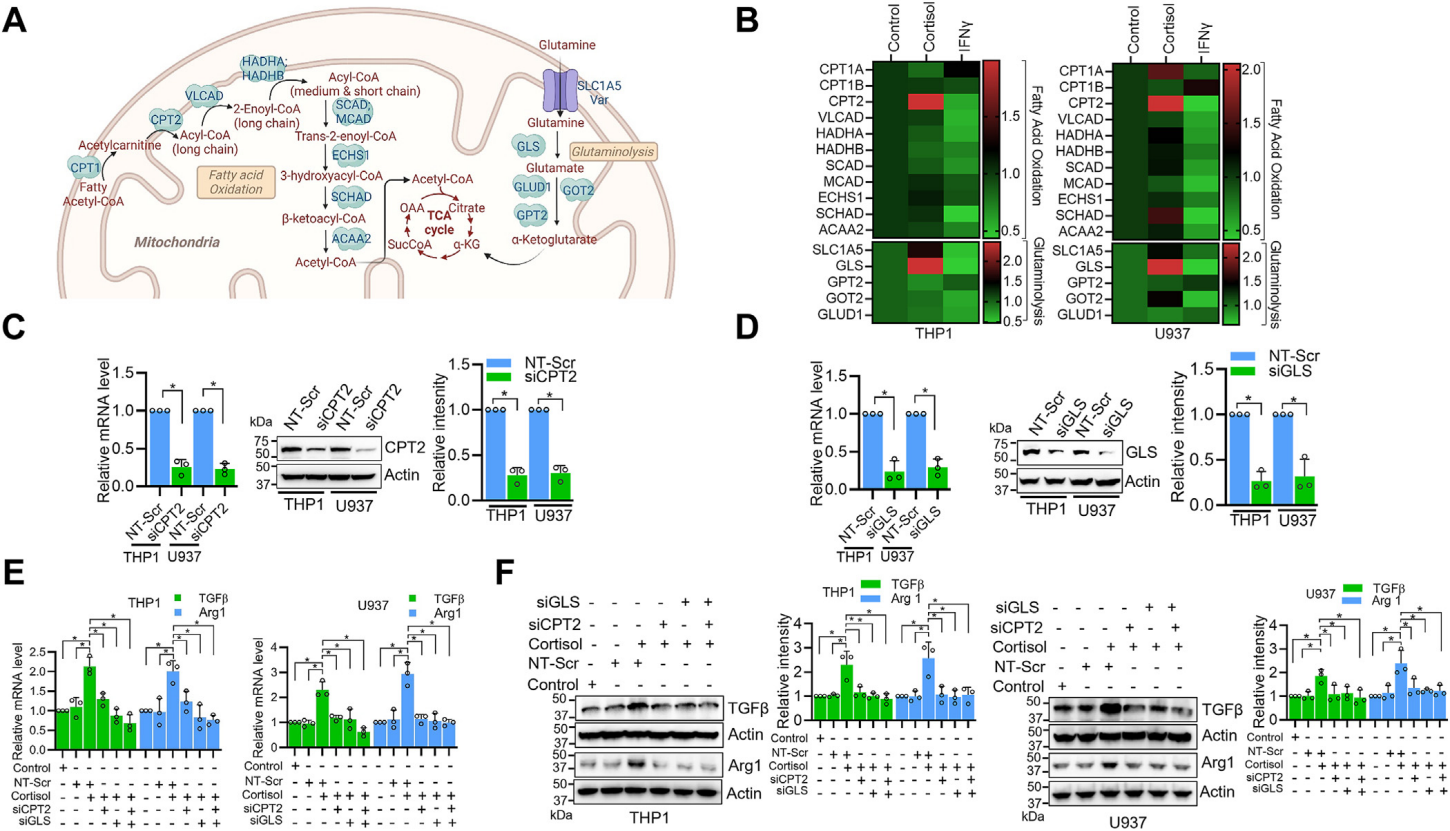
**Cortisol upregulates *CPT2* and *GLS* to sustain OXPHOS through TCA anaplerosis *via* enhanced FAO and glutaminolysis pathways.***A*, Graphical illustration showing enzymes involved in TCA anaplerosis through FAO and glutaminolysis pathways. *B*, qRT-PCR-based gene expression analysis of enzymes involved in FAO and glutaminolysis pathways in THP1- and U937-derived macrophages treated for 48 h with cortisol or IFNγ. *C*, siRNA-mediated knockdown of *CPT2* in THP1- and U937-derived macrophages as analyzed by qRT-PCR and western blotting. *D*, siRNA-mediated knockdown of *GLS* in THP1- and U937-derived macrophages analyzed by qRT-PCR and western blotting. *E*–*F*, analysis of M2 marker (TGFβ and Arg1) expression in THP1-derived macrophages-treated with cortisol, CPT2 siRNA with cortisol, GLS siRNA with cortisol, or both siRNAs with cortisol by qRT-PCR (*E*) and Western blot analysis (*F*). Bars in panels *C*, *D*, and *F* represent quantitative data from three biological replicates. Data were subjected to unpaired Student’s *t* test (*C*, *D*) or One-Way ANOVA with Tukey’s multiple comparisons test (*E*, *F*) for statistical significance. ∗*p* < 0.05.

To understand how OXPHOS is sustained in cortisol-treated macrophages despite reduced glycolysis, we focused our attention on fatty acid oxidation (FAO) and glutaminolysis, known for TCA anaplerosis (Fig. 7*A*). We earlier observed that cortisol increased the dependency of macrophages on fatty acid and glutamine to maintain OXPHOS (Fig. S8*B*). Therefore, we examined the expression of genes involved in these metabolic pathways in cortisol-treated THP1- and U937-derived macrophages. We found that cortisol induced the expression of *CPT2* encoding carnitine palmitoyltransferase 2 responsible for the conversion of acetylcarnitine to long-chain acyl-CoA in fatty acid oxidation pathway and *GLS* which codes for glutaminase that catalyzes the conversion of glutamine to glutamate in glutaminolysis pathways. At the same time, these genes were suppressed by IFNγ (Fig. 7*B*). This data and previous observations indicate that M1 macrophages primarily depend on the glycolysis pathway and not on the alternate pathways to feed the TCA cycle. To further explore if FAO and glutaminolysis are crucial for M2 macrophage polarization, we downregulated the expression of *CPT2* and *GLS* by using specific siRNAs and examined the effect of cortisol treatment in these cells. The data show effective downregulation of *CPT2* and *GLS* by siRNAs (Fig. 7, *C* and *D*). Further, cortisol treatment fails to induce M2 polarization in *CPT2* and *GLS* downregulated cells suggesting an important role of fatty acid oxidation and glutaminolysis in cortisol-induced macrophage M2 polarization. The effect was higher in *GLS* downregulated macrophages, while M2 polarization was completely inhibited when cells were treated with both *CPT2* and *GLS* siRNAs prior to cortisol treatment (Fig. 7, *E* and *F*).

## Discussion

Our study has revealed a novel mechanism through which cortisol confers its immunosuppressive action. We have found that cortisol induces the expression of miR-143/miR-145 cluster in macrophages, which then alters glucose metabolism to affect their polarization state. MicroRNAs have emerged as a novel class of gene regulators playing important roles in maintaining cellular homeostasis (30, 31, 32). Consequently, aberrant expression of miRNAs is associated with various pathophysiological conditions such as cancer, heart diseases, neurodegenerative diseases, diabetes, *etc*. (33, 34, 35, 36). It has been estimated that an individual miRNA molecule possesses the capability to target approximately 200 distinct transcripts (37). Depending on the target gene transcript, miRNAs modulate processes like cell signaling, cellular differentiation, inflammation, energy metabolism, immune evasion, chemoresistance, angiogenesis, *etc*. (38, 39, 40, 41, 42, 43).

The regulation of miRNA expression occurs at the transcriptional and post-transcriptional processing levels such as pri-miRNA processing to mature miRNA by pri-miRNA editing (44, 45, 46). It is also shown that the changes in the cellular environment can modulate the expression of miRNAs through altered cell signaling (47, 48). Macrophages are highly plastic and acquire a polarization state depending upon the external stimuli from the surrounding environment. It has been shown that the infection of macrophages with *mycobacterium* induces miR-155 expression, which reduces the nitric oxide-dependent anti-mycobacterial activity. Transfecting the macrophages with miR-155 inhibitor leads to the rescue of nitric oxide synthesis, thus reducing mycobacterial burden (49). Interestingly, in another report, LPS-induced miR-146a has been shown to downregulate IL-6 production in macrophages suggesting that it may have a broader impact through stromal remodeling (50). Altered expression of miR-142-3p, miR-30b, and miR-24 during monocyte-to-macrophage differentiation is also shown to regulate the phagocytic activity of macrophages as well as the release of cytokines (51). Along these lines, our findings showing induction of miR-143-3p and miR-145-5p by cortisol are significant in understanding how the hormonal imbalance under stress could affect macrophage function through miRNA expression.

Classically activated macrophages (M1) could be activated by IFNγ released by Th1 cells or NK cells or by exposure to lipopolysaccharide, a component of the bacterial membrane (52, 53). Similarly, exposure of macrophages to IL-4 secretion from Th2 cells or cancer cells results in their alternate activation or M2 polarization (52, 54, 55). It is shown that the metabolic alterations accompany these functional and phenotypic changes in macrophages. The M1 macrophages rely more on glycolytic metabolism than M2 macrophages causing an enhanced production of NADPH, which is then utilized by enzymes, such as NADPH oxidase, resulting in the production of reactive oxygen species and reactive nitrogen species to neutralize the pathogens (56, 57). These alterations also induce the synthesis and secretion of inflammatory cytokines (58, 59). On the contrary, M2 macrophages rely primarily on OXPHOS sustained through TCA anaplerosis *via* FAO and glutamine metabolism (60). However, what triggers these metabolic changes in the macrophages has not been completely understood. In that regard, our findings bring novel insight by demonstrating the role of miR-143-3p and miR-145-5p in altered glucose metabolism through the negative regulation of *HK2* and *ADPGK*, respectively. This is interesting as in another study, tumor-derived exosomal miRNA let-7a is shown to affect insulin-Akt-mTOR pathway increasing OXPHOS metabolism and M2 polarization under hypoxia conditions (61). Thus, it appears that macrophages can employ different mechanisms to affect their polarization state.

*HK2* catalyzes the formation of glucose-6-phosphate from glucose by utilizing ATP as a source for phosphate transfer to glucose, whereas *ADPGK* uses ADP as a phosphate donor (62, 63). It has been reported that *HK2* expression in immune cells is positively correlated with higher immune cell infiltration in the tumor microenvironment (TME) (64). *HK2* is also required for bacterial peptidoglycan detection by macrophages (65), and the functions of the microglial (66) and activated T-cells (67). It is also required for the production of inflammatory cytokines by macrophages (68). Genome-wide analysis has suggested the predominant expression of *ADPGK* in hematopoietic lineages, such as macrophages, T and B cells, dendritic cells, and monocytes (29). It is involved in T-cell activation by enhancing glycolysis (69). These prior observations along with our findings show that *HK2* and *ADPGK* expression is required for immune cell activation and certainly for the M1 polarization of the macrophages.

The glycolysis in M1 polarized macrophages is crucial for pro-inflammatory phenotypes, phagocytosis, secretion of inflammatory cytokines, and ROS production (56, 59, 70). The TCA cycle in M1 macrophages has two break points resulting in the accumulation of itaconic acid and succinic acid. Conversely, the M2 macrophages have a functional TCA cycle and are more dependent on OXPHOS (56). Our data show that although cortisol reduces the glycolysis through microRNA-mediated repression of *HK2* and *ADPGK*, OXPHOS maintenance and M2 polarization depended on cortisol-induced expression of FAO pathway enzyme, CPT2, and glutaminolysis pathway enzyme, GLS. Another important enzyme of the glutaminolysis pathway, glutamine synthase, has been shown to prime macrophage polarization towards the M2 phenotype (71). Glucocorticoid was shown to induce the expression of glutamine synthase in osteoblastic cells and human leukemic cells (72, 73).

**Figure 8:**
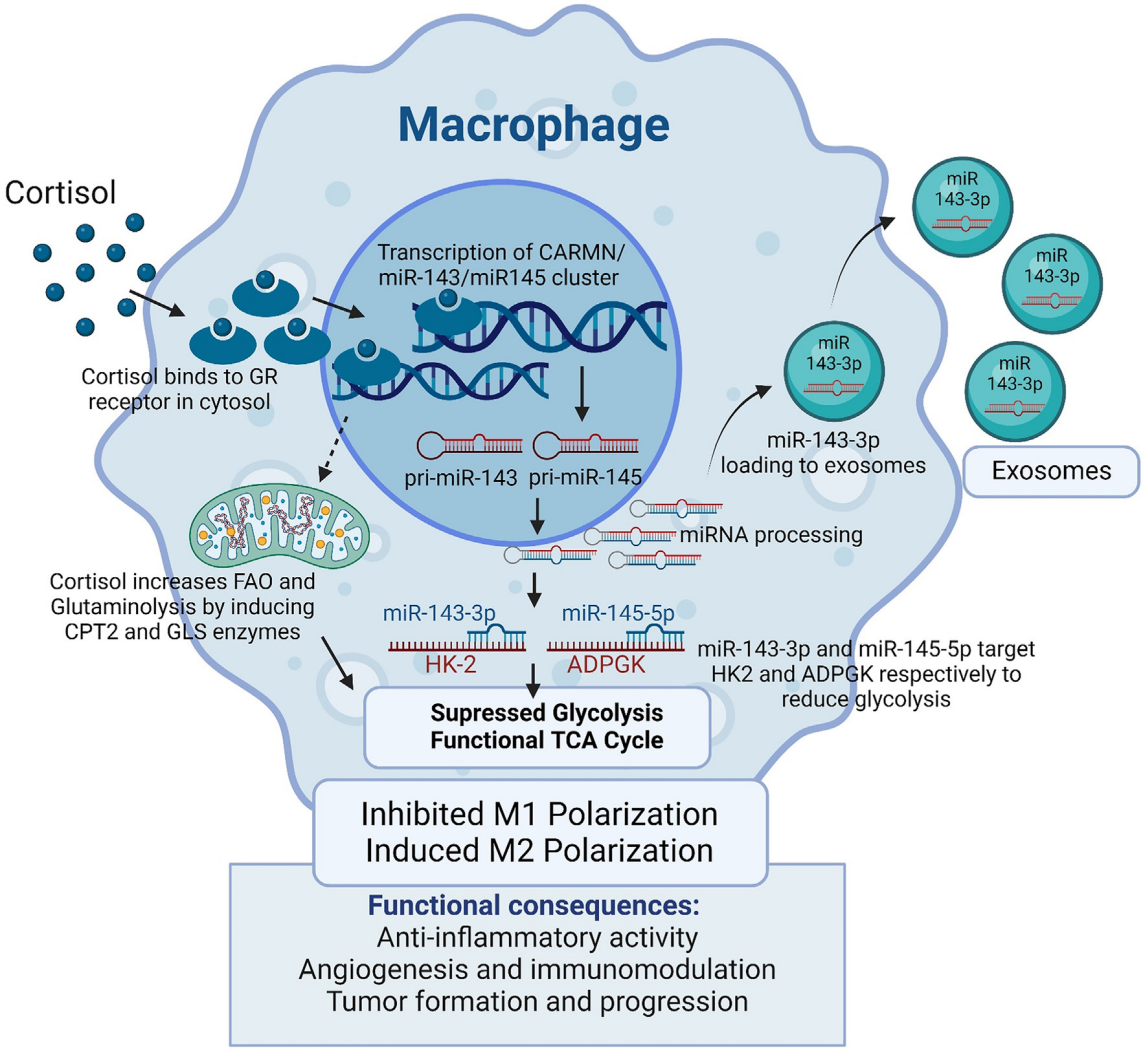
**Schematic summary depicting the effect of cortisol on macrophage polarization and implicated molecular mechanisms.** Cortisol affects the polarization state of macrophages through transcriptional up-regulation of miR-143-3p and miR-145-5p, which target *HK2* and *ADPGK*, respectively, to reprogram glucose metabolism. Cortisol also increases TCA anaplerosis by upregulating fatty acid oxidation and glutaminolysis through upregulation of *CPT2* and *GLS*, respectively. miR-143-3p, but not miR-145-5p, is loaded into exosomes and released into the extracellular spaces and thus can have wider implications in human physiology and disease development.

Altogether, our study has unfolded a novel mechanism for macrophage polarization in response to the exposure of cortisol, a major stress hormone (Fig. 8). Since TME comprises multiple other cell types, it is important to examine if cortisol also affect these cells to gain a broader understanding. Further, it is also imperative to examine the role of CARMN lncRNA in macrophage phenotype. As a host gene for miR-143 and miR-145, *CARMN* is reported to be crucial for maintaining the contractile and mobility phenotype of muscle cells (74, 75). However, a direct role of CARMN lncRNA in macrophages or any other non-muscle cell type has not yet been elucidated. Realization of stress-generated changes in immune cell metabolism and function is vital to developing an improved understanding of stress-related pathologies, including dementia and cancer. Therefore, efforts to minimize stress could positively impact human health and improve the clinical outcomes of patients.

## Experimental procedures

All the chemicals, cell lines, antibodies, siRNA, miRNA mimics, miRNA inhibitors, commercial assay kits, and other reagents for this study were procured from different sources. The product catalog number and other details are provided in Tables S2–S4.

### Blood collection and isolation of peripheral blood mononuclear cells (PBMC) and serum

This study was approved by the Institutional Review Board (IRB-1143193) and performed in accordance with the Declaration of Helsinki. Blood samples were collected from human subjects after obtaining their written consent and subjected to PBMC and serum isolation. For PBMC isolation, the blood samples were diluted with Dulbecco’s Phosphate Buffered Saline (DPBS) containing 2% FBS (Stemcell Technologies) in 1:1 volume ratio. Two volumes of diluted blood were gently layered on top of a one volume lymphoprep solution (Stemcell Technologies) and centrifuged at 800*g* for 20 min. PBMCs were harvested from the liquid interface. Serum was isolated as previously described by us (76).

### Cell culture

The human monocytes (THP1 and U937) and BC cell lines (MDA-MB-468 and MDA-MB-231) were purchased from the American Type Culture Collection (ATCC) and cultured in RPMI and DMEM media, respectively (Corning) at 37 °C in a humidified incubator with 5% CO_2_. The cell culture medium was supplemented with heat-inactivated Fetal Bovine Serum (FBS; 10%; R&D Systems) and penicillin/streptomycin antibiotic (1×; Gibco). Short tandem repeats genotyping and intermittent testing for cell line-specific marker proteins were performed to validate the authenticity of the cell lines. To obtain M0 macrophages, THP1 and U937 cells were treated with Phorbol 12-myristate 13-acetate (PMA; 20 ng/ml; 24 h; Millipore Sigma) and were followed by a rest period (24 h) in culture medium. For monocyte culture from human subject-derived PBMCs, harvested PBMCs were washed with 1× PBS and plated in monocyte attachment medium (Sigma Aldrich) for 1.5 h in six-well culture plates. The media was aspirated to remove non-adherent cells and adherent cells were subjected to vigorous swirling in a warm monocyte attachment medium to remove any weakly adhered cells. The adhered monocytes were then incubated for 6 days in macrophage generation medium XF (Sigma Aldrich) containing 30 ng/ml M-CSF.

### Exosome isolation

The exosomes were isolated from cultured cells using a total exosome isolation reagent (Invitrogen) and processed according to the manufacturer's protocol (77). Briefly, the cell culture medium was collected after 48 h of treatment and centrifuged for 30 min at 2000*g* to remove the cell debris and processed for exosome isolation. Exosomes from the serum of BC patients were isolated from 250 μl samples using SmartSEC Single EV Isolation System (SBI System Biosciences) according to the manufacturer protocol.

### Treatments

For M1 polarization, the M0 macrophages were treated with recombinant human IFNγ (50 ng/ml; 48 h; R&D Systems), and for M2 polarization, the M0 macrophages were treated with recombinant human IL-4 (50 ng/ml; 48 h; R&D Systems) in a culture medium. Cortisol (200 ng/ml; Millipore Sigma) was used for experimental treatments. All treatments were performed in a medium supplemented with charcoal-stripped FBS (10%; Gibco) and penicillin-streptomycin antibiotic solution (1×; Gibco). Cell lines were periodically tested for *mycoplasma* contamination using the MycoAlert *Mycoplasma* Detection Kit (Lonza).

### Enzyme-linked immunosorbent assay (ELISA)

To determine the cortisol levels in serum samples of BC patients, the human serum cortisol ELISA kit (R&D systems) was used according to the manufacturer's protocol. Optical density was measured at 450 nm using a BioTek microplate reader (Agilent). Unknown concentrations were calculated using the standard curve.

### RNA extraction and quantitative real-time PCR (qRT-PCR)

Total RNA from cells was isolated using the mirVana miRNA isolation kit (Invitrogen) according to the manufacturer’s protocol. RNA was quantified using Nanodrop 1000 (Thermo Scientific). Total RNA from exosomes was isolated using the Total Exosome RNA & Protein Isolation Kit (Invitrogen). For miRNA expression analysis, first total RNA was subjected to reverse transcription using a high-capacity cDNA reverse transcription kit (Applied Biosystems). The miRNA reverse transcription primer containing a stem-loop extension in addition to the complementary miRNA sequence was used as described earlier (78). For mRNA expression analysis, 2 μg total RNA was reverse transcribed using random primers. The quantitative real-time PCR (qRT-PCR) was performed using Maxima SYBR Green/ROX qPCR master mix (Thermo Scientific) on a CFX96 real-time PCR system (Bio-Rad). Fold change in miRNA expression was calculated using the ΔΔCt method. Actin and U6 Ct were used to normalize the data for mRNA and miRNA respectively. To analyze expressions of pri-miR-143, pri-miR-145, and *CARMN*, a pre-designed TaqMan PCR assay was used (Applied Biosystems) using U6 and actin as an internal control respectively. Primer sequences that were used are provided in Table S2.

### Chromatin-immunoprecipitation (ChIP) assay

The binding of GRα to the *CARMN* promoter region was confirmed using the ChIP-IT Express Enzymatic kit (Active Motif) as described earlier (79). Briefly, the protein was cross-linked to DNA in a living cell with formaldehyde. The cells were lysed, and DNA was enzymatically sheared. Immunoprecipitation was conducted with an anti-GRα antibody (Abcam) or IgG control using protein G magnetic beads. The immunoprecipitated DNA was amplified by qRT-PCR for *CARMN* promoter regions (P1:727–562; P2:1441–1282; P3: 4981–4797). Fold change of ChIP DNA was determined using qRT-PCR. Primer sequences are provided in Table S2.

### Dual luciferase assay

For promoter reporter assay, pEZX-GA01 vector (GeneCopoeia) containing wild-type or mutant *CARMN* promoter regions was cloned upstream of secretory Gaussia luciferase and was transfected in THP1- and U937-derived M0 macrophages using X-treme Gene HP DNA transfection reagent (Roche Diagnostics). After 24 h of transfection, cells were treated with 200 ng/ml cortisol for wild-type or mutant *CARMN* promoter induction. For 3′ UTR-reporter assay, pEZX-MT05 vector (GeneCopoeia) containing 3′ UTR region of *HK2* or *ADPGK* gene was cloned downstream of secretory Guassia luciferase and was transfected in THP1- and U937-derived M0 macrophages using X-treme Gene HP DNA transfection reagent (Roche Diagnostics). After 24 h of vector DNA transfection, cells were either treated with 200 ng/ml cortisol or transfected with miR-143-3p or miR-145-5p mimic. The culture medium from both assays was collected post-cortisol treatment (48 h) and analyzed for luciferase activity using a secrete-pair dual luminescence assay kit (GeneCopoeia). Secreted alkaline phosphatase (SeAP) was used as an internal control to measure transfection efficiency.

### Flow cytometry analysis

The THP1 and U937 cells were counted and seeded in 12-well plate in equal numbers with 20 ng/ml PMA. Cell media was changed with fresh RPMI media (Corning) after 24 h. After 48 h of seeding, the cells were treated according to the required experimental conditions. The attached cells were harvested using a macrophage detachment solution (Sigma Aldrich) as per manufacturer instructions. The harvested cells were washed one time with stain buffer (BD biosciences) by centrifugation at 1200 rpm for 5 min and resuspended in 100 μl stain buffer. The cells were incubated on ice with human Fc Block reagent (BD Biosciences) for 20 min. Fluorophore-labeled target-specific antibodies were added according to the experimental requirement and incubated for 30 min at RT in the dark. The cells were then washed three times with stain buffer (BD Biosciences) by centrifugation at 1200 rpm for 5 min each. The percentage of stained cells was determined by flow cytometry analysis using BD FACSCanto II (BD Biosciences).

### siRNA, miRNA mimic, and miRNA inhibitor transfection

Knockdown of *HK2*, *ADPGK*, *CPT2* and *GLS* was performed using ON-TARGETplus HK2 siRNA-SMART pool, ON-TARGETplus ADPGK siRNA-SMART pool, ON-TARGETplus CPT2 siRNA-SMART pool and ON-TARGETplus GLS siRNA-SMART pool (Dharmacon, Lafayette, CO, USA), respectively. siGenome non-target siRNA pool was used as a negative control (Dharmacon). The miRNA mimic and miRNA inhibitors for miR-143-3p and miR-145-5p (Life technologies) were used to mimic or inhibit miRNA functions. The 50 nM of siRNAs, miRNA mimics or miRNA inhibitors were transfected using lipofectamine RNAi max reagent (Invitrogen). Cells were seeded a day before transfection to get approximately 70% confluency. 50 μl of Opti-MEM I (Invitrogen) was taken in two separate tubes per well for transfection. 5 μl lipofectamine RNAi max reagent was added to the first tube, and siRNA, mimic or inhibitor for 50 nM final concentration was added to the second tube containing Opti-MEM I per well for transfection. The content of the two vials was then mixed and incubated for 15 min at room temperature. Meanwhile, the medium of the seeded cells was changed to a fresh RPMI complete medium followed by the gentle addition of a transfection mix. Cells were placed at 37 °C in a CO_2_ incubator and fresh media was added after 24 h. The treated cells then proceeded for RNA and protein isolation after 48 h of transfection.

### Protein extraction and immunoblotting

The total cell protein was extracted using ice-cold RIPA buffer (Thermo Scientific) and was estimated using a DC protein assay kit (Bio-Rad). For Western blot, 60 to 80 μg of total denatured protein with loading buffer were separated on SDS-PAGE gel and transferred to the polyvinylidene fluoride membrane (PVDF; Bio-Rad). The membrane was blocked using 5% skimmed milk in 1× PBST for 1 h. The blocked membrane was incubated with the antibody against the target proteins at 1:1000 dilution in 5% milk-PBST overnight at 4 °C. Washing was performed using PBST (3X) for 5 to 10 min each. HRP conjugated secondary antibody was added onto the membrane at 1:1000 in 5% milk-PBST and incubated for 60 to 90 min. After secondary antibody incubation, the membrane was washed (3X) with PBST for 5 to 10 min each. SuperSignal West Femto Maximum sensitivity substrate kit (Thermo Scientific) was used to visualize protein bands. Protein bands were imaged using ChemiDoc System (Bio-Rad) and band intensity was determined using Image J software.

### Extracellular acidification rate and oxygen consumption rate assay

Extracellular acidification and oxygen consumption rate (ECAR and OCR) of THP1 and U937 cells were assessed using Seahorse XFe96 Analyzer according to the manufacturer’s protocol using the Agilent Seahorse XF Glycolysis Stress and Mito Stress Test Kits (Agilent Technologies), respectively. The ECAR and OCR experiments were performed in a predefined seahorse XF RPMI assay medium (Agilent Technologies), pH 7.4, supplemented with HEPES buffer to stabilize the pH. In brief, 8 × 10^4^ (THP1) and 3 × 10^4^ (U937) cells/well were plated for M0 differentiation in Seahorse XF96 Cell Culture Microplates (Agilent Technologies) in PMA (20 ng/ml; 24 h) containing medium, followed by a rest period (24 h) in PMA free medium. After 48 h, M0 macrophages were treated with IFNγ for M1 polarization, IL-4 for M2 polarization, and cortisol for ECAR and OCR levels. For ECAR, after baseline measurement in glucose-free media, at indicated time points, the glucose, oligomycin (ATP synthase inhibitor), and 2-DG (glycolysis inhibitor) were sequentially injected with real-time measurements for extracellular acidification. For OCR, oligomycin, mitochondrial membrane decoupler FCCP (p-trifluoromethoxy carbonyl cyanide phenylhydrazone), and mitochondrial complex I and III inhibitors rotenone plus antimycin A (Rot/AA) were sequentially injected for real-time measurement for oxygen consumption. Data was analyzed using Seahorse Wave desktop software. Cell numbers were used to normalize the data. The final ECAR values are presented in mpH/min, and OCR values are presented in pmol/min.

### Fuel flexibility assay

The XF Mito Fuel Flex Test Kit (Agilent Technologies) was used to determine the fuel flexibility and dependency on glucose, glutamine, or fatty acid to feed the TCA cycle using Seahorse XFe96 Analyzer (Agilent Technologies), as per manufacturer’s protocol. The THP1 (8 × 10^4^) and U937 (3 × 10^4^) cells/well were plated and differentiated to M0 macrophages with PMA treatment. M0 cells were then treated with IFNγ for M1 polarization, IL-4 for M2 polarization, and cortisol to compare the fuel dependency and flexibility of the cells. As per the protocol, UK5099 (inhibitor of the glucose oxidation pathway), BPTES (inhibitor of the glutamine oxidation pathway), and etomoxir (inhibitor of long-chain fatty acid oxidation) were injected into the wells. Flue dependency and capacity were calculated using Seahorse Wave desktop software. Cell numbers were used to normalize the data.

### Lactate assay

Lactate assay was performed in THP1- and U937-derived macrophages to determine the changes in glycolysis upon M1 or M2 induction. An equal number of cells were seeded in 6-well plate with PMA induction for 24 h followed by 24 h rest period. These M0 macrophages were then treated with 50 ng/ml IFNγ, 50 ng/ml IL-4, or 200 ng/ml cortisol for 48 h. Culture media was harvested and lactate estimation was performed using L-lactate assay kit (Abcam) according to the manufacturer protocol. The lactate values were normalized to the number of cells in each well counted post treatment.

### Statistical analysis

The quantitative data derived from three independent biological replicates are reported as mean ± SD in bar or line diagrams. Statistical comparisons between two groups were performed by unpaired Student's *t* test and for multiple group comparisons, ordinary one-way ANOVA was used. Pearson correlation coefficient was used to determine the association between serum cortisol levels and exosomal miRNAs. All statistical analyses were performed using GraphPad Prism 9 (GraphPad Software Inc). A *p* value of lower than 0.05 was considered statistically significant.

## Data availability

The data supporting the study is available from the corresponding author upon request.

## Supporting information

This article contains supporting information.

## Conflict of interest

The authors declare that they have no conflicts of interest with the contents of this article.
