# Supplementary material for: Cortisol affects macrophage polarization by inducing miR-143/145 cluster to reprogram glucose metabolism and by promoting TCA cycle anaplerosis
Source: J Biol Chem. 2024 Sep 10;300(10):107753. doi: 10.1016/j.jbc.2024.107753 (PMC11470657; doi:10.1016/j.jbc.2024.107753)
Supplement: Supporting information [file mmc1.docx]

**Cortisol affects macrophage polarization by inducing miR-143/145 cluster to reprogram glucose metabolism and by promoting TCA cycle anaplerosis**

Amod Sharma^1,2^, Kunwar Somesh Vikramdeo^1,2^, Sarabjeet Kour Sudan^1,2^, Shashi Anand^1,2^, Sachin Kumar Deshmukh^3,4,5^, Ajay Pratap Singh^1,2^, Seema Singh^1,2,*^

^1^Cancer Center and Research Institute, University of Mississippi Medical Center, Jackson, MS 39216

^2^ Department of Cell and Molecular Biology, University of Mississippi Medical Center, Jackson, MS 39216

^3^Mitchell Cancer Institute, University of South Alabama, Mobile, AL 36604

^4^Department of Pathology, University of South Alabama, Mobile, AL 36617

Present address: ^5^ Caris Life Sciences, Phoenix, AZ, 85040

This study was supported by the National Institutes of Health /National Cancer Institute (CA205297) and the University of South Alabama Mitchell Cancer Institute.

*Address correspondence and reprint requests to:

Seema Singh, PhD, MBA

Cancer Center and Research Institute

Department of Cell and Molecular Biology

University of Mississippi Medical Center

2500 North State Street, Jackson, MS 39216.

E-mail: [ssingh3@umc.edu](mailto:ssingh3@umc.edu)

**Running Title: Cortisol affects macrophage polarization.**


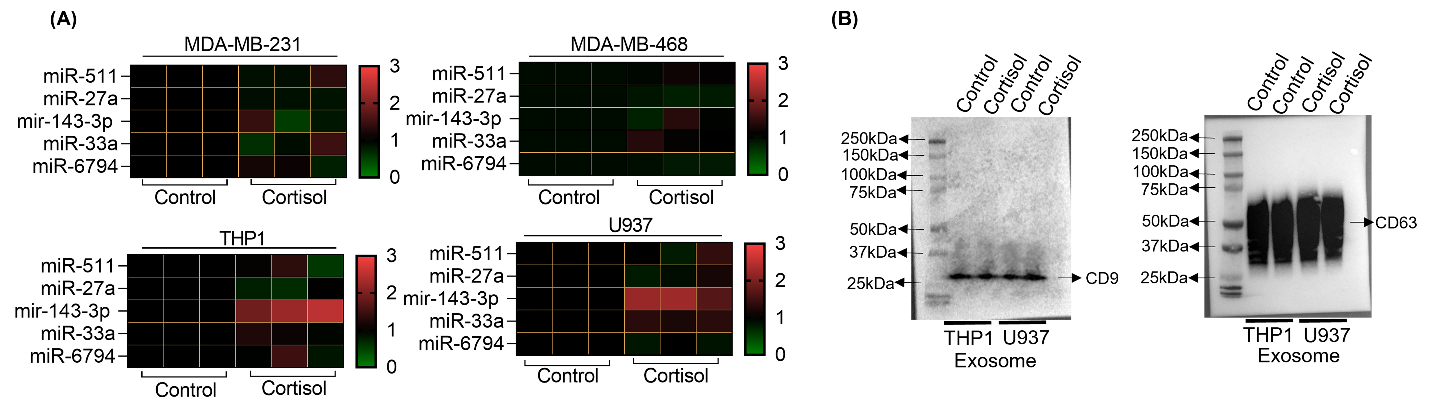


**Supplementary Figure S1:** (A) Heat map showing expression profiling of miR-511, miR-27a, miR-143-3p, miR-33a and miR-6794 in breast cancer cell lines (MDA-MB-231 and MDA-MB-468) and monocyte cell lines THP1- and U937-derived macrophages following treatment with either control or cortisol (100 ng/ml) for 24 h. (B) Western blot data showing the expression of marker proteins (CD9 and CD63) in exosomes isolated from THP1- and U937-derived macrophages treated with control or 200 ng/ml cortisol for 48 h.


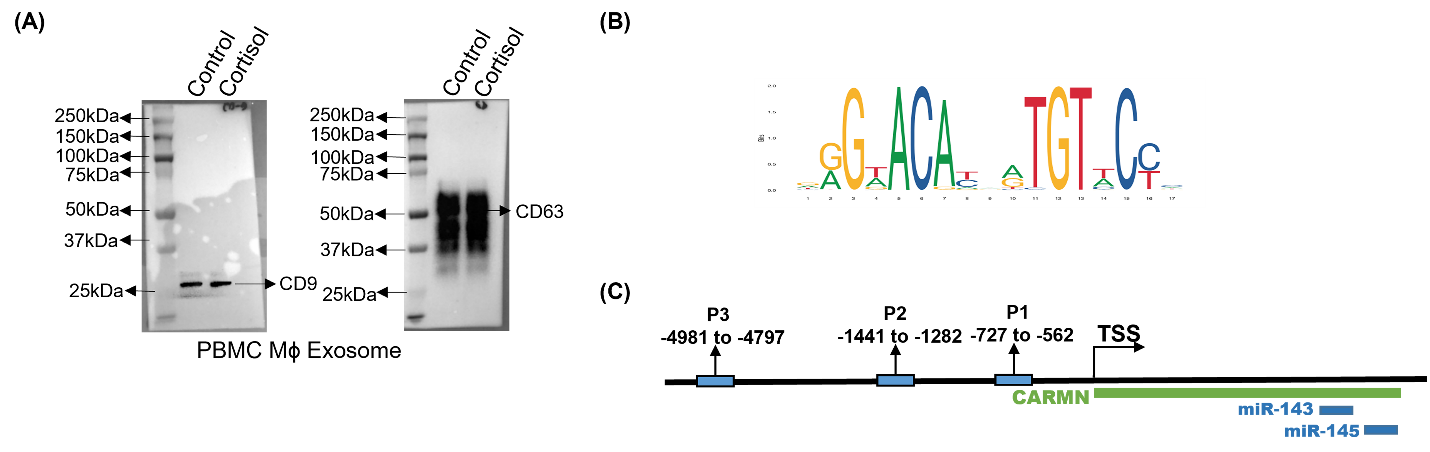


**Supplementary Figure S2:** (A) Western blotting analysis shows expression of exosomal markers CD9 and CD63 in exosomes isolated from PBMC-derived macrophages following treatment with control or 200 ng/ml cortisol for 48 h. (B) Consensus sequence of glucocorticoid response element predicted by JASPAR database. (C) Graphical illustration showing GRα binding sites predicted in CARMN promoter region using JASPAR database.


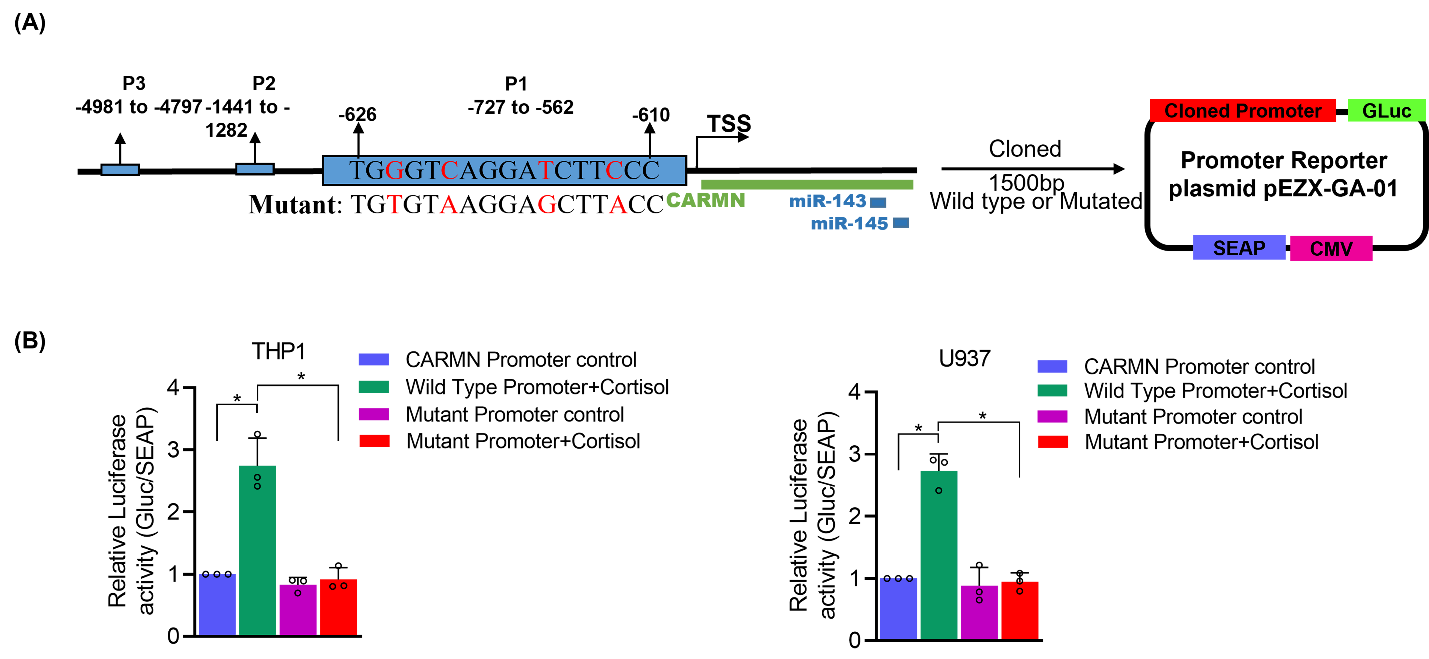


**Supplementary Figure S3:** (A) Graphical illustration showing glucocorticoid response element site in CARMN promoter region and mutated nucleotide (shown in red) sequence cloned in pEZX-GA-01 vector for luciferase-based promoter reporter assay. (B) Reduction in CARMN promoter activity with mutated glucocorticoid response element in THP1- and U937-derived macrophages analyzed by luciferase promoter reporter assay. Statistical analysis was performed using unpaired *t*-test. *p <0.05 was considered to be statistically significant.


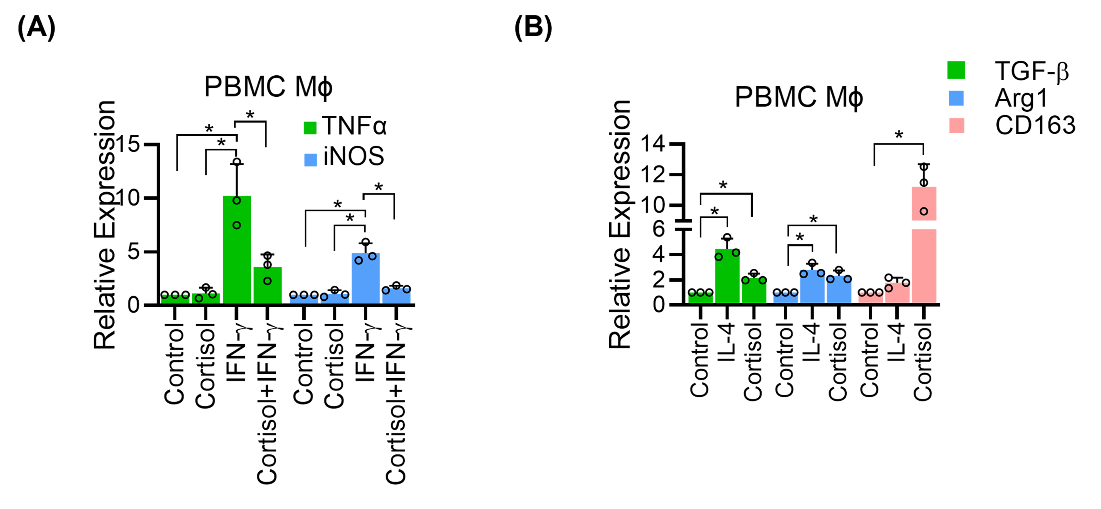


**Supplementary Figure S4:** (A) qRT-PCR data showing inhibition of IFNγ-induced M1 polarization markers (TNFα and iNOS) by cortisol pretreatment in PBMC-derived macrophages. (B) qRT-PCR data showing induction of M2 polarization markers (TGFβ, Arg1, and CD-163) upon treatment with 200 ng/ml cortisol or 50 ng/ml IL-4 in PBMC-derived macrophages. Statistical significance was determined via Tukey’s multiple comparisons test following ordinary One-Way ANOVA. *p <0.05 was considered to be statistically significant.


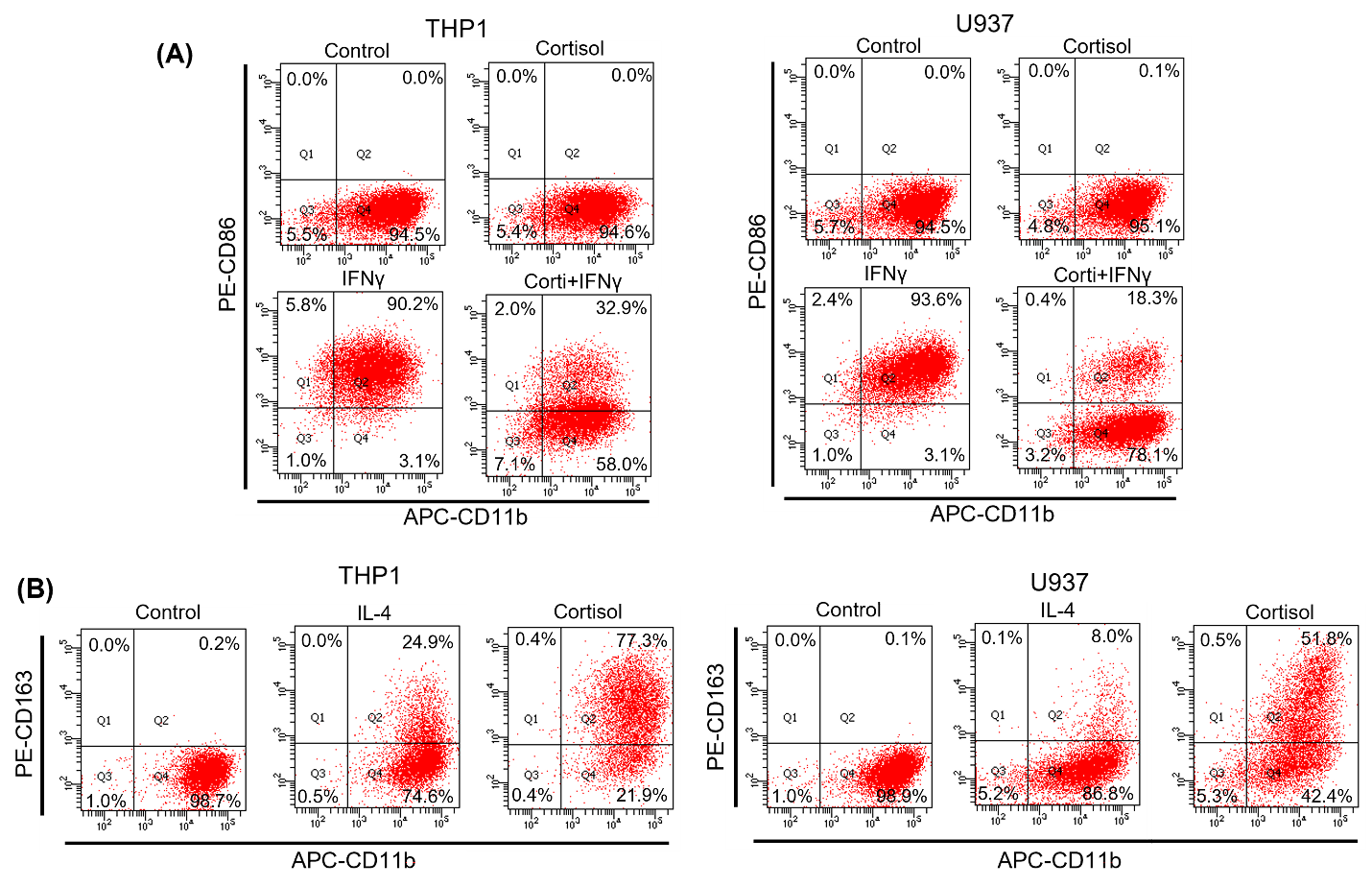


**Supplementary Figure S5:** (A) Flow cytometry analysis showing inhibition of IFNγ (50 ng/ml)-induced M1 polarization marker (CD86) by cortisol (200 ng/ml) pretreatment in THP1- and U937-derived macrophages analyzed after 48 h treatment. CD11b represents the marker for total macrophages. (B) Flow cytometry analysis showing induction of M2 polarization markers (CD-163) upon 48 h treatment with 200 ng/ml cortisol or 50 ng/ml IL-4 in THP1- and U937-derived macrophages. CD11b was used as marker for total macrophages.


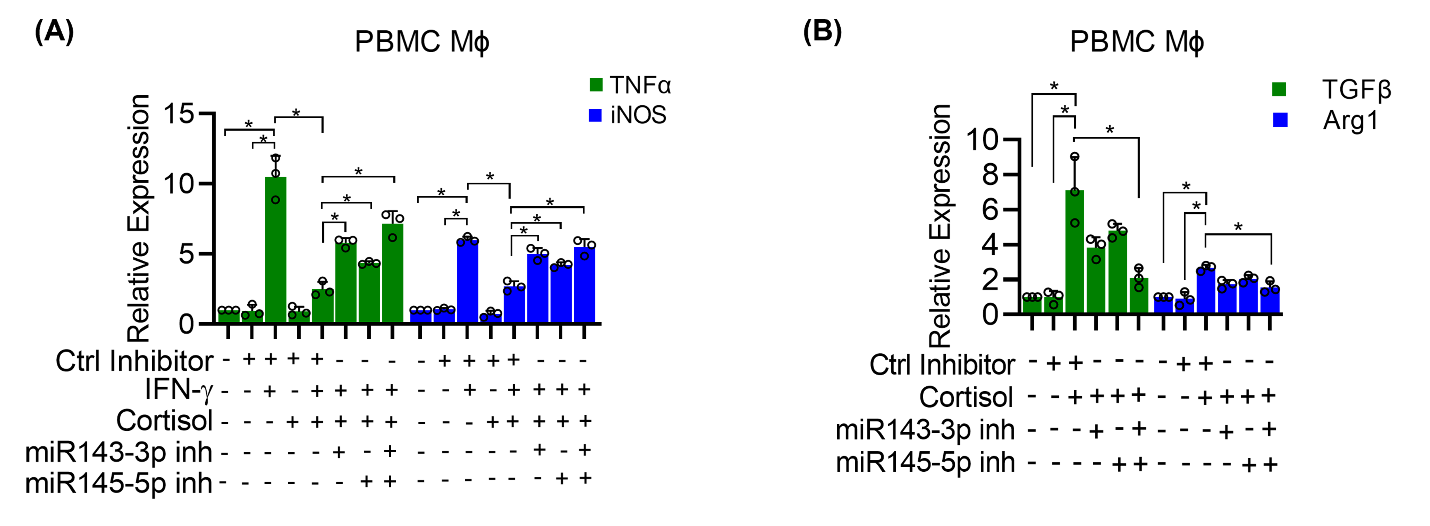


**Supplementary Figure S6:** (A) qRT-PCR data showing the rescue of IFNγ (50 ng/ml) -induced M1 polarization inhibition caused by cortisol (200 ng/ml) pretreatment, using miRNA inhibitors (50 nM) for miR-143-3p or/and miR-145-5p in PBMC-derived macrophages. (B) qRT-PCR data showing expression of M2 polarization markers TGFβ and Arg1, representing inhibition of cortisol (200 ng/ml)- induced M2 polarization, using miRNA inhibitors (50 ng/ml) for miR-143-3p and miR-145-5p in PBMC-derived macrophages. *p <0.05 was considered to be statistically significant.


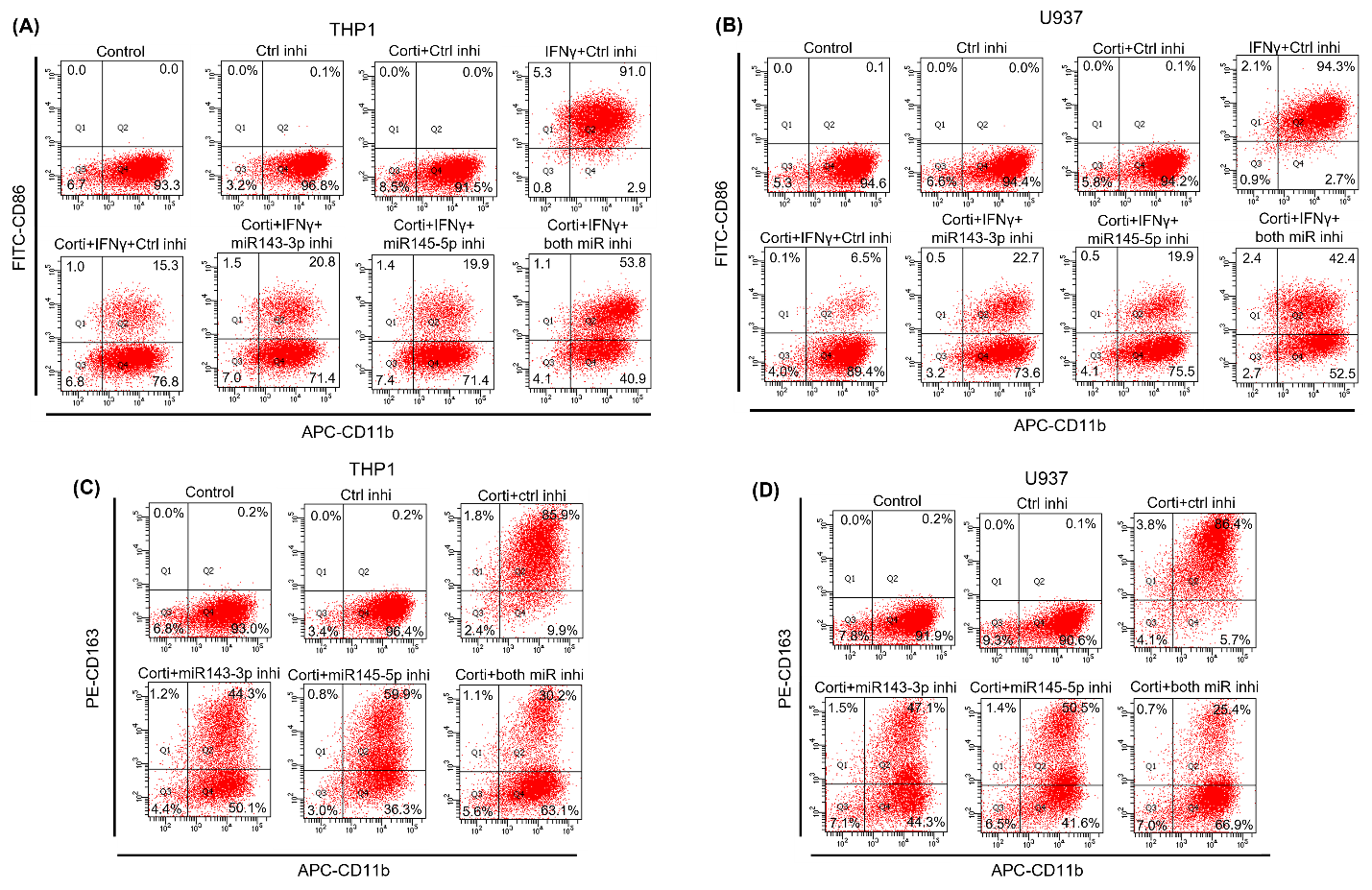


**Supplementary Figure S7:** (A and B) Flow cytometry analysis showing the rescue of IFNγ (50 ng/ml) -induced M1 polarization inhibition caused by cortisol (200 ng/ml) pretreatment, using miRNA inhibitors for miR-143-3p or/and miR-145-5p in THP1- and U937-derived macrophages. CD86 was used as M1 polarization marker and CD11b as total macrophage marker. (C and D) Flow cytometry analysis showing the inhibition of cortisol (200 ng/ml)- induced M2 polarization, using miRNA inhibitor for miR-143-3p and miR-145-5p in THP1- and U937-derived macrophages, analyzed after 48 h of treatment, CD163 was used as M2 polarization marker and CD11b as total macrophage marker.


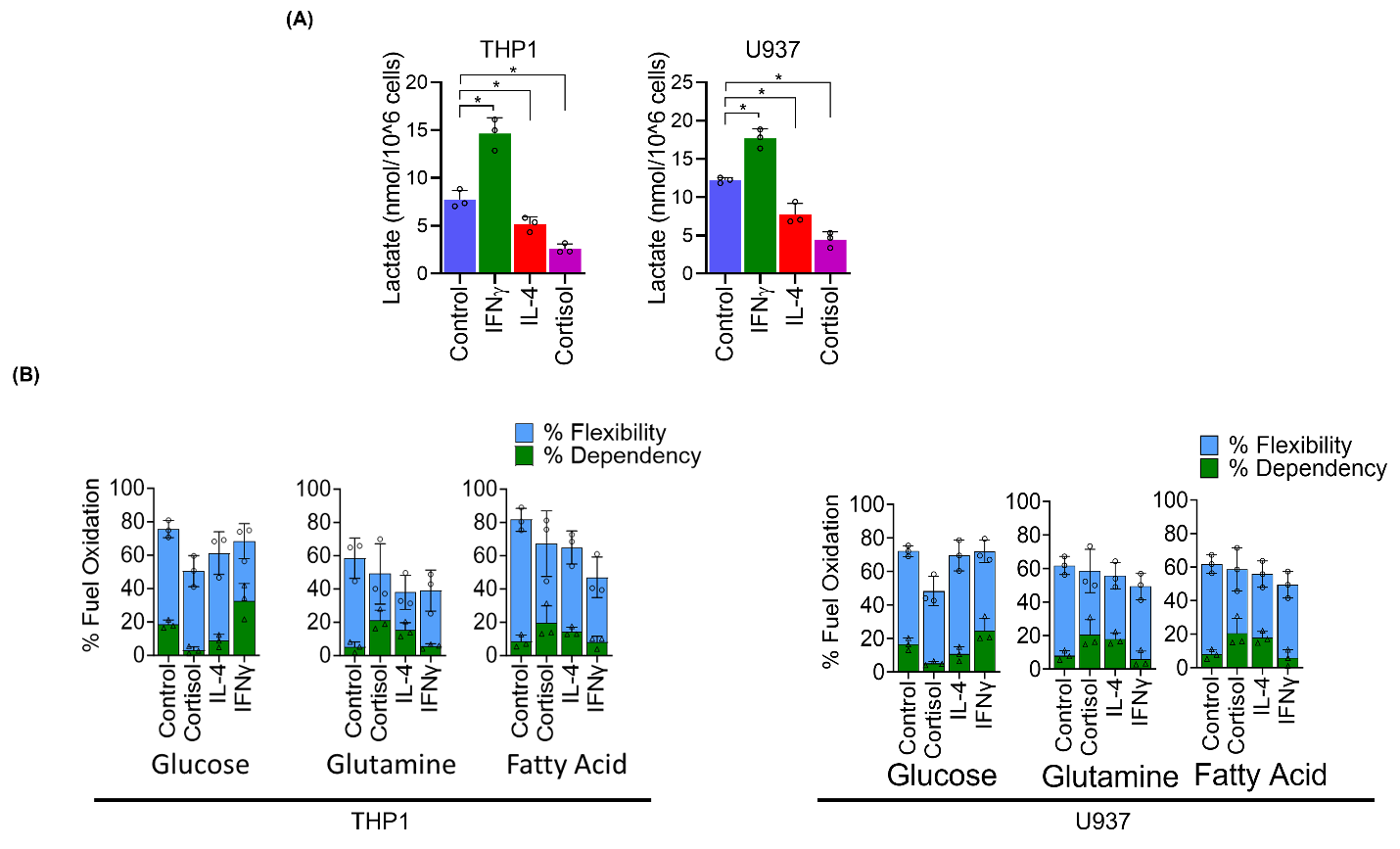


**Supplementary Figure S8:** Lactate assay (A) showing level of glycolysis and Fuel flexibility assay (B) representing glutamine and fatty acid fuel flexibility and dependency for OXPHOS in THP1- and U937-derived macrophages treated with either control or IFNγ (50 ng/nl) or IL-4 (50 ng/ml) or Cortisol (200 ng/ml) for 48 h.


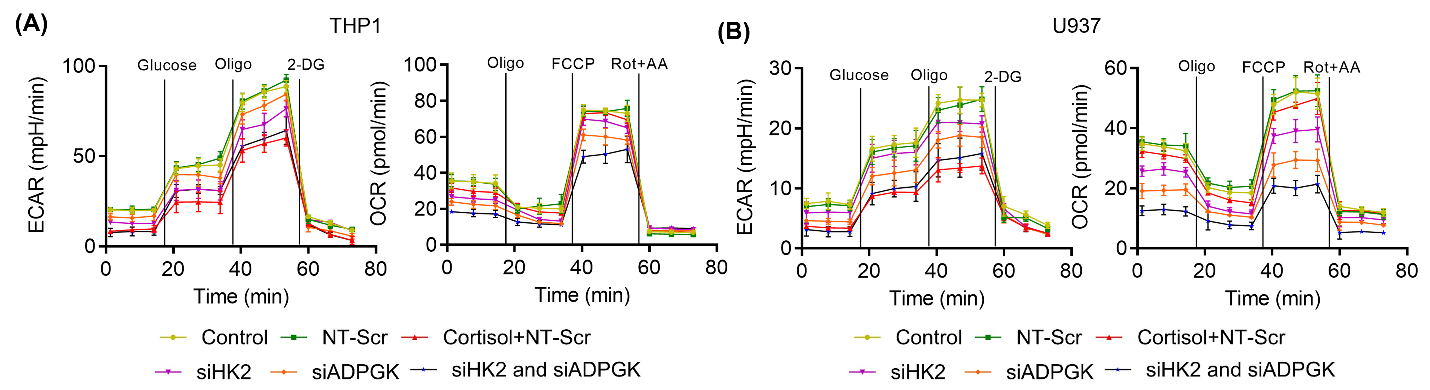


**Supplementary Figure S9:** (A and B) ECAR and OCR assay representing glycolysis and OXPHOS levels respectively, in THP1- (A) and U937- (B) derived macrophages treated with either control or cortisol, or siHK2 or siADPGK or both siRNAs for 48 h.

**Supplementary Table S1:** Ct value of miRNAs determined by real time PCR from macrophages treated with either control or cortisol.

| Approx. Ct value in 1µg RNA | | | | |
| --- | --- | --- | --- | --- |
|  | Control | | Cortisol | |
|  | THP1 | U937 | THP1 | U937 |
| miR-143-3p | 29.537 | 30.831 | 27.474 | 28.725 |
| mirR-145-5p | 27.711 | 26.366 | 25.849 | 25.238 |
| miR-143-5p | 36.689 | 36.337 | 36.881 | 36.449 |
| miR-145-3p | 37.555 | 36.786 | 37.114 | 37.040 |

**Supplementary Table S2:** List of reagents and kits used in the study with their source and catalog numbers.

| **Reagents and Kits** | **Source** | **Identifier** |
| --- | --- | --- |
| RPMI (Roswell Park Memorial Institute) | Corning, AZ, USA | Cat# 10-013-CM |
| DMEM (Dulbecco's Modified Eagle Medium) | Corning, AZ, USA | Cat# 10-013-CV |
| FBS (Fetal Bovine Serum) | R&D Systems, MN, USA | Cat# S11150H |
| Penicillin-Streptomycin (10,000 U/mL) | Gibco, NY, USA | Cat# 15140122 |
| Trypsin-EDTA (0.25%) | Gibco, NY, USA | Cat# 25200-056 |
| Macrophage base medium XF | Sigma Aldrich, MO, USA | Cat# C-28057 |
| Monocyte attachment medium | Sigma Aldrich, MO, USA | Cat# C-28051 |
| Lymphoprep | Stemcell Technologies, MA, USA | Cat# 07851 |
| DPBS with 2% FBS | Stemcell Technologies, MA, USA | Cat# 07905 |
| PMA (Phorbol 12-myristate 13-acetate) | Millipore Sigma, MA, USA | Cat# P8139 |
| Recombinant human IFNγ | R&D Systems, MN, USA | Cat# 285-IF-100 |
| Recombinant human IL-4 | R&D Systems, MN, USA | Cat# 204-IL-010 |
| Recombinant human M-CSF | R&D Systems, MN, USA | Cat# 216-MC-005 |
| Cortisol | Millipore Sigma, MA, USA | Cat# C-106 |
| mirVana miRNA isolation kit | Invitrogen, Vilnius, Lithuania | Cat# AM1561 |
| High capacity cDNA reverse transcription kit | Applied Biosystems, MA, USA | Cat# 4368813 |
| Maxima SYBR green/ROX qPCR master mix | Thermo Scientific, MA, USA | Cat# K221 |
| Agilent Seahorse XF Glycolysis Stress Test Kit | Agilent Technologies, CA, USA | Cat# 103020-100 |
| Agilent seahorse XF Mito Stress test kit | Agilent Technologies, CA, USA | Cat# 103015-100 |
| XF Mito Fuel Flex Test Kit | Agilent Technologies, CA, USA | Cat# 103270-100 |
| Pierce RIPA buffer | Thermo Scientific, IL, USA | Cat# 89900 |
| DC protein assay | Bio-Rad, CA, USA | Cat# 5000111 |
| PVDF membrane | Thermo Scientific, IL, USA | Cat# 88518 |
| SuperSignal West Femto Maximum sensitivity substrate kit | Thermo Scientific, MA, USA | Cat# 34095 |
| ChIP-IT Express Enzymatic kit | Active Motif, CA, USA | Cat# 53009 |
| X-treme Gene HP DNA transfection reagent | Roche Diagnostics, IN, USA | Cat# 6366236001 |
| Secrete-pair dual luminescence assay kit | GeneCopoeia, MD, USA | Cat# LF032 |
| Lipofectamine RNAi max reagent | Invitrogen, CA, USA | Cat# 13778030 |
| Opti-MEM-I | Invitrogen, CA, USA | Cat# 31985062 |
| Total Exosome Isolation Reagent | Invitrogen, CA, USA | Cat# 4478359 |
| SmartSEC Single EV Isolation System | SBI system biosciences | Cat# SSEC200A-1 |
| Total Exosome RNA and Protein Isolation kit | Invitrogen, CA, USA | Cat# 4478545 |
| Cortisol ELISA kit | RD Systems, MN, USA | Cat# KGE008B |
| mirVANA miRNA mimic  has-miR-143-3p | Ambion by Life Technologies, MA, USA | Cat# MC10883 |
| mirVANA miRNA mimic  has-miR-145-5p | Ambion by Life Technologies, MA, USA | Cat# MC11480 |
| mirVANA miRNA inhibitor  has-miR-143-3p | Ambion by Life Technologies, MA, USA | Cat# MH10883 |
| mirVANA miRNA inhibitor  has-miR-145-5p | Ambion by Life Technologies, MA, USA | Cat# MC11480 |
| ON-Targetplus Human HK-2 siRNA-SMARTpool | Dharmacon, CO, USA | Cat# L-006735-00-0005 |
| ON-Targetplus Human ADPGK siRNA-SMARTpool | Dharmacon, CO, USA | Cat# L-006809-00-0005 |
| ON-Targetplus Human GLS siRNA-SMARTpool | Dharmacon, CO, USA | Cat# L-004548-01-0005 |
| ON-Targetplus Human CPT2 siRNA-SMARTpool | Dharmacon, CO, USA | Cat# L-008574-00-0005 |
| siGENOME Non-Targeting siRNA pool | Dharmacon, CO, USA | Cat# D-001206-13-05 |
| Human BD Fc block | BD Biosciences, NJ, USA | Cat# 564220 |
| L-Lactate Assay Kit | Abcam, MA, USA | Cat# ab169557 |

**Supplementary Table S3:** List of antibodies used in study with their supplier and catalog numbers.

| **Antibody** | **Source** | **Identifier** |
| --- | --- | --- |
| HK-2 | Cell Signaling | Cat# 2106S |
| ADPGK | Abcam | Cat# ab228633 |
| TNF-α | Novus | Cat# NBP1-19532 |
| iNOS | Novus | Cat# NB300-605 |
| TGF-β | Thermo Fisher Scientific | Cat# MA5-15065 |
| Arg1 | Abcam | Cat# ab133543 |
| GRα | Novus | Cat# NB300-633 |
| CD-163 | Abcam | Cat# ab182422 |
| Actin | Cell Signaling | Cat# 4970L |
| GLS | Novus Biologicals | Cat# NBP1-89766 |
| CPT2 | Novus Biologicals | Cat# NBP3-15294 |
| CD63 | Cell Signaling | Cat# 52090S |
| CD9 | Thermo Fisher Scientific | Cat# 0626D |
| APC-CD11b | BD Biosciences | Cat# 550019 |
| FITC-CD86 | BD Biosciences | Cat# 555657 |
| PE-CD163 | BD Biosciences | Cat# 567881 |

**Supplementary Table S4:** List of primers with their sequence used in the study.

| **S. No.** | **Primer name** | **Sequence (5̍-3̍)** |
| --- | --- | --- |
| **1.** | miR-143-3p RT | GTCGTATCCAGTGCAGGGTCCGAGGTATTCGCACTGGATACGACGAGCTA |
| **2.** | miR-143-3p F | TCGGCGTGAGATGAAGCACTG |
| **3.** | miR-145-5p RT | GTCGTATCCAGTGCAGGGTCCGAGGTATTCGCACTGGATACGACAGGGAT |
| **4.** | miR-145-5p F | TCGGCGGTCCAGTTTTCCCAGGA |
| **5.** | miR-143-5p RT | GTCGTATCCAGTGCAGGGTCCGAGGTATTCGCACTGGATACGACACCAGAG |
| **6.** | miR-143-5p F | TCGGCGGGTGCAGTGCTGCAT |
| **7.** | miR-145-3p RT | GTCGTATCCAGTGCAGGGTCCGAGGTATTCGCACTGGATACGACAGAACAG |
| **8.** | miR-145-3p F | TCGGCGGGATTCCTGGAAATA |
| **9.** | miR-511-5p RT | GTCGTATCCAGTGCAGGGTCCGAGGTATTCGCACTGGATACGACTGACTG |
| **10.** | miR-511-5p F | TCGGCGGTGTCTTTTGCTCTG |
| **11.** | miR-27a-5p RT | GTCGTATCCAGTGCAGGGTCCGAGGTATTCGCACTGGATACGACTGCTCA |
| **12.** | miR-27a-5p F | TCGGCGAGGGCTTAGCTGCTTG |
| **13.** | miR-33a-5p RT | GTCGTATCCAGTGCAGGGTCCGAGGTATTCGCACTGGATACGACTGCAAT |
| **14.** | miR-33a-5p F | TCGGCGGTGCATTGTAGTTGC |
| **15.** | miR-6794-5p RT | GTCGTATCCAGTGCAGGGTCCGAGGTATTCGCACTGGATACGACGCTCAC |
| **16.** | miR-6794-5p F | CGCAGGGGGACTGGGG |
| **17.** | Reverse primer for miR | GTCGTATCCAGTGCAGGGTCCGAGGT |
| **18.** | HK-2 F | GAGTTTGACCTGGATGTGGTTGC |
| **19.** | HK-2 R | CCTCCATGTAGCAGGCATTGCT |
| **20.** | CD163 F | CCAGAAGGAACTTGTAGCCACAG |
| **21.** | CD163 R | CAGGCACCAAGCGTTTTGAGCT |
| **22.** | ADPGK F | ATGGAGGGACAAAGCAAGGAGC |
| **23.** | ADPGK R | GCTCCCTGTTAGTCATACTGGC |
| **24.** | Actin F | CACCATTGGCAATGAGCGGTTC |
| **25.** | Actin R | AGGTCTTTGCGGATGTCCACGT |
| **26.** | iNOS F | GCTCTACACCTCCAATGTGACC |
| **27.** | iNOS R | CTGCCGAGATTTGAGCCTCATG |
| **28.** | TNF-α F | CTCTTCTGCCTGCTGCACTTTG |
| **29.** | TNF-α R | ATGGGCTACAGGCTTGTCACTC |
| **30.** | TGF-β F | TACCTGAACCCGTGTTGCTCTC |
| **31.** | TGF-β R | GTTGCTGAGGTATCGCCAGGAA |
| **32.** | Arg1 F | TCATCTGGGTGGATGCTCACAC |
| **33.** | Arg1 R | GAGAATCCTGGCACATCGGGAA |
| **34.** | CARMN promoter P1 F | GAGATGGGCATTTCTCAGGA |
| **35.** | CARMN promoter P1 R | GTGGGAAGCTACTCCCATCA |
| **36.** | CARMN promoter P2 F | CCACTCAGGATGTCACAAGG |
| **37.** | CARMN promoter P2 R | GTCTCTGTTCACGGCCTCAT |
| **38.** | CARMN promoter P3 F | AATTCCCTCCACCCAGAGAT |
| **39.** | CARMN promoter P3 R | TTCCTGCTATCACCAGCACA |
| **40.** | GLS F | CAGAAGGCACAGACATGGTTGG |
| **41.** | GLS R | GGCAGAAACCACCATTAGCCAG |
| **42.** | CPT2 F | GCAGATGATGGTTGAGTGCTCC |
| **43.** | CPT2 R | AGATGCCGCAGAGCAAACAAGTG |
